# Mechanism and deterioration pattern of sandstone surrounding rock voiding at bottom of heavy-haul railway tunnel

**DOI:** 10.1038/s41598-024-61149-5

**Published:** 2024-05-04

**Authors:** Zi-qiang Li, Shi-jian Yang, Jian-wen Feng, Hang Zhang, Wei-wei Huang, Zheng Li

**Affiliations:** 1 School of Civil Engineering and Architecture, Chongqing University of Science and Technology, Chongqing, 401331 China; 2 School of Resources and Safety Engineering, Chongqing University, Chongqing, 400045 China; 3 Chongqing City Construction Investment (Group) Co., Ltd., Chongqing, 400023 China; 4 Chongqing Energy Investment Group Co., Ltd., Chongqing, 401120 China; 5 Agiletech Engineering Consultants Co., Ltd., Beijing, 100050 China

**Keywords:** Heavy-haul railway tunnel, Sandstone surrounding rock, Voiding mechanism, Deterioration patterns, Discrete element method, Civil engineering, Petrology

## Abstract

This study combines laboratory experiments and discrete element simulation methods to analyze the mechanism and deterioration patterns of sandstone surrounding rock voiding the bottom of a heavy-haul railway tunnel. It is based on previously acquired measurement data from optical fiber grating sensors installed in the Taihangshan Mountain Tunnel of the Wari Railway. By incorporating rock particle wastage rate results, a method for calculating the peak strength and elastic modulus attenuation of surrounding rock is proposed. Research indicates that the operation of heavy-haul trains leads to an instantaneous increase in the dynamic water pressure on the bottom rock ranging 144.4–390.0%, resulting in high-speed water flow eroding the rock. After 1–2 years of operation, the bottom water and soil pressures increase by 526.5% and 390.0%, respectively. Focusing on sandstone surrounding rock with high observability, laboratory experiments were conducted to monitor the degradation stages of infiltration, particle loss, and voiding of rock under the action of dynamic water flow. The impact of water flow on the “cone-shaped” bottom rock deformation was also clarified. The extent of rock deterioration and voiding was determined using miniature water and soil pressure sensors in conjunction with discrete element numerical simulations. The measured rock particle loss was used as a criterion. Finally, a fitting approach is derived to calculate the peak strength and elastic modulus attenuation of surrounding rock, gaining insight into and providing a reference for the maintenance and disposal measures for the bottom operation of heavy-haul railway tunnels.

## Introduction

Heavy-haul railways have been widely recognized and valued by countries worldwide because of their high capacity, efficiency, low energy consumption, and transportation costs. Because heavy-haul railways are characterized by high axle load, high total weight, and high traffic density, they are prone to damage in tunnel structures. Among the various types of tunnel damage, the sinking and upheaval of tunnel bottom account for the highest proportion. The former is primarily caused by inadequate compaction, exacerbating structural damage at the bottom of the tunnel. Moreover, field investigations reveal that the bottom sinking of sections in heavy-haul railway tunnels are typically accompanied by abundant groundwater. The combined effects of train loads and groundwater in heavy-haul railway tunnels are presumed to influence the state of surrounding rock at the bottom, thereby compromising the safety of tunnel structures and accelerating structural damage. Therefore, the conduct of an in-depth study on the voiding mechanism and deterioration patterns of sandstone surrounding rock at the bottom of heavy-haul railway tunnels is necessary.

Yang et al.^1^ employed numerical simulation methods to establish a three-dimensional dynamic numerical model of train loads, tunnels, and surrounding rock and concluded that the cyclic loading–unloading effect of train loads has a significant impact on void width. Hua et al.^2^ employed numerical simulation and calculation methods to analyze the effect of void-induced changes on the contact area between the surrounding rock and arch on the tunnel contact pressure. The study found that the worse the condition of surrounding rock, the more significant the impact of bottom void development on the tunnel structure. Using finite element simulation, Xue^3^ found that groundwater was the primary cause of uneven distribution in weak soil layers. Inadequate foundation reinforcement and vibrations from train loads during operation were the key factors that accelerated voiding in these layers. Zhang et al.^4^ conducted numerical analysis to investigate the influence of soil voiding on tunnel stability in a layer comprising sandstone and pebbles. They explored the evolutionary patterns of voiding damage in surrounding rock. Dai^5^ employed a two-dimensional discrete element method to analyze the voiding process at the bottom surrounding rock under the influence of heavy-haul train loads. The voiding conditions were assessed based on the particle displacement of the bottom surrounding rock and changes in porosity. Wang et al.^6^ combined a damage model with an elastoplastic model to simulate the stress evolution and energy transfer mechanism during hard rock failure. This approach yielded the shapes of stress–strain curves for various stages and forms of rock failure. The foregoing shows that most current studies focus on the causes of voiding of the lining structure. However, research is limited on the damage and voiding process of the bottom surrounding rock in heavy-haul railway tunnels under the combined influence of inherent defects, heavy-haul train actions, and groundwater. Furthermore, several research methods are based on qualitative predictive analyses using continuous-medium numerical simulations, data surveys, and theoretical derivations. Quantitative research on the degree of deterioration and voiding of rock surrounding a tunnel bottom using field measurements and laboratory experiments is lacking.

This study aims to explore the mechanism and deterioration patterns of voiding at the bottom surrounding rock of a heavy-haul railway tunnel. Based on the measured data of Wari Railway Taihangshan Mountain Tunnel, laboratory experiments were conducted on selected sandstone surrounding rock using a self-designed experimental setup. An indoor experimental model was established to simulate the voiding characteristics and formation processes of the surrounding sandstone. By studying the defect locations, defect severity, water and soil pressure values, and particle loss at the bottom surrounding rock, the voiding mechanism of the bottom surrounding rock under dynamic water flow was observed. After clarifying the void formation process at the bottom surrounding rock of heavy-haul railway tunnels, the particle flow code (PFC) particle flow discrete element model was applied. This model was used to elucidate the forms of particle detachment and loss under the action of dynamic water flow and to determine the deterioration patterns and voiding range of surrounding rock. Finally, the influence of the surrounding rock particle wastage rate on the attenuation of mechanical parameters is deduced, enabling the derivation of corresponding attenuation formulas.

## Engineering and field measurements

### Project overview

**Figure 1:**
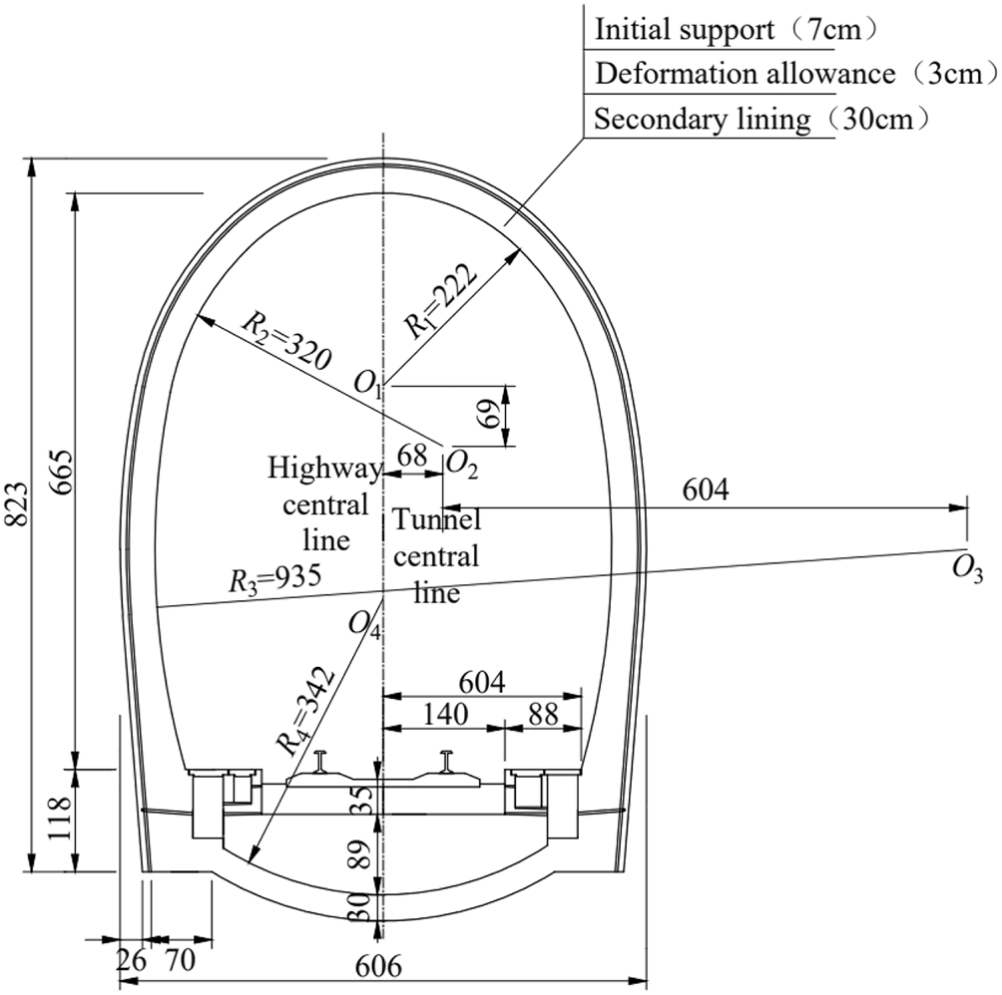
Cross-section of Taihangshan mountain tunnel lining.

Based on a study of the Taihangshan Mountain Tunnel of the Wari Railway, preliminary monitoring of the water and soil pressures in the tunnel was conducted. An unballasted trackbed structure was adopted for the entire tunnel. The Taihangshan Mountain Tunnel is a double-track, single-line, heavy-haul railway tunnel. It passes through three types of surrounding rock conditions—Classes III, IV, and V—with the majority of the areas being water-rich environments. The selected test area is the Class V surrounding rock section; the lining cross-section is shown in Fig. 1.

Sandstone, clay, and pebbles are distributed in the selected area. Of these, sandstone is the most widely distributed, and widely distributed sandstone typically appears as sediment. Accordingly, sandstone possesses intermediate properties between those of clay and pebbles, combining the characteristics of the three materials. The relatively comprehensive nature of sandstone provides a suitable basis for the experimental analyses. Therefore, in this study, sandstone surrounding rock is selected for the experimental analysis.

### Long-term monitoring program

#### Location of measurement points

**Figure 2:**
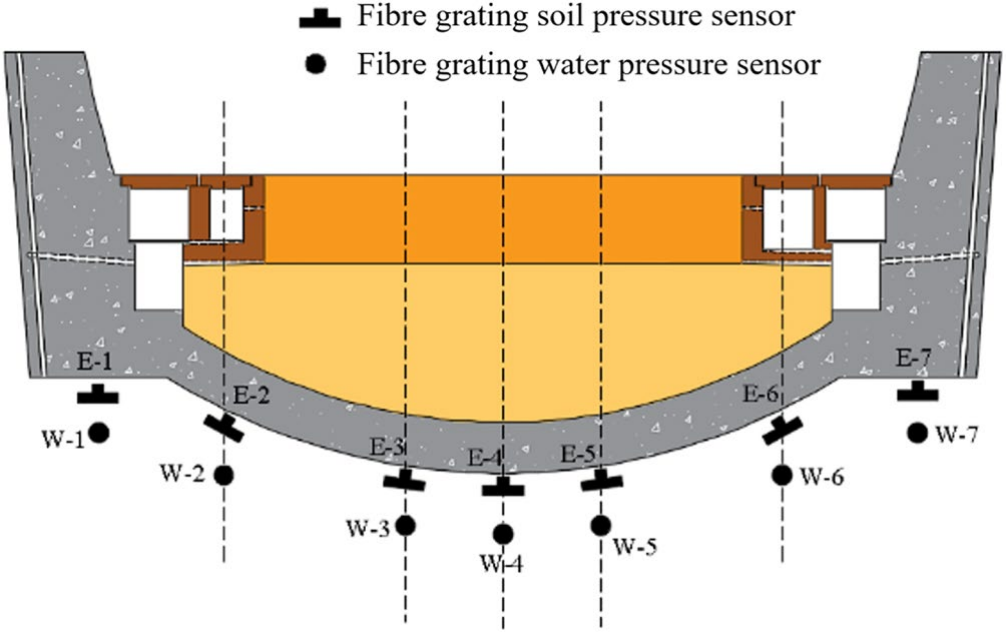
Schematic of layout of sensor in surrounding rock of bedrock.

During the initial construction phase of the Taihangshan Mountain Tunnel, fiber grating water pressure and soil pressure sensors were symmetrically embedded on the surface of surrounding rock at the test section. To investigate the combined effects of train dynamic loads and groundwater on the characteristics and positions of the bottom structure, measurement points are selected at the crown, vertical side ditch, vertical track, and centerline positions, as shown in Fig. 2.

The actual operating parameters of the heavy-haul train in the Taihangshan Mountain Tunnel were axle load and speed of 300 kN and 80 km/h, respectively. The monitoring system was configured to activate data collection as the heavy-haul train passed through the test section; the data collection interval was 0.01 s.

### Test sensor

**Figure 3:**
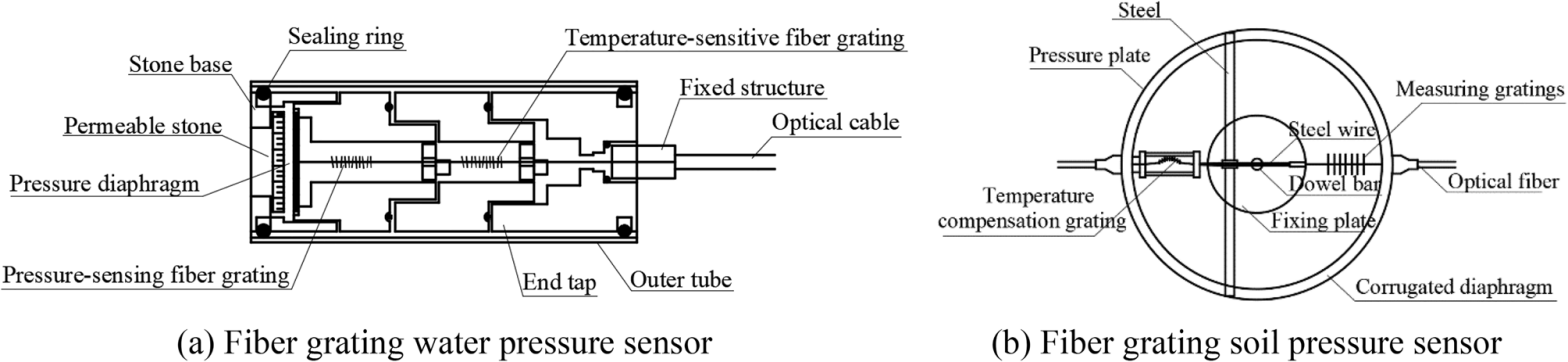
Fiber grating water and soil pressure sensor structure schematic.

**Figure 4:**
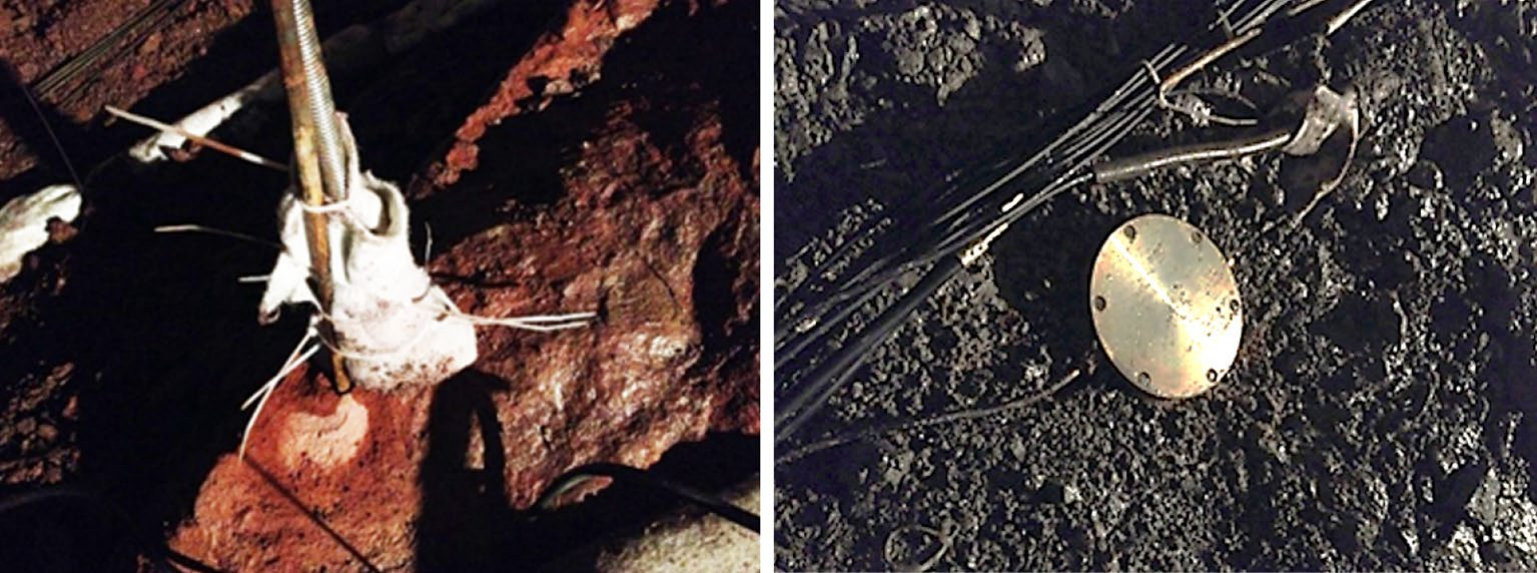
Fiber grating water and soil pressure sensor installation diagram.

Given the complex hydrogeological conditions of the Taihangshan Mountain Tunnel, the test sensors chosen must meet the requirements for strong anti-interference, long-term durability, and stable test data. Therefore, robust fiber grating water and soil pressure sensors are used, as shown in Fig. 3. The measuring range of the soil pressure sensors (E-1–E-7) was up to 2 MPa, whereas that for the water pressure sensors (W-1–W-7) was up to 700 kPa. The installation of sensors is shown in Fig. 4.

### Remote water and soil pressure measurement results

Based on the on-site monitoring data, each passage of a heavy-haul train through the monitoring section evidently exerts a cumulative effect on the water and soil pressures at the bottom surrounding rock. Remote measurements revealed significant long-term variations in water and soil pressures. The total number of water pressure measurement points on the bottom surrounding rock at the site is 7; symmetric points exhibit similar dynamic water pressure time history curves. Therefore, only the time history curves of measured water pressure on one side are presented.

**Figure 5:**
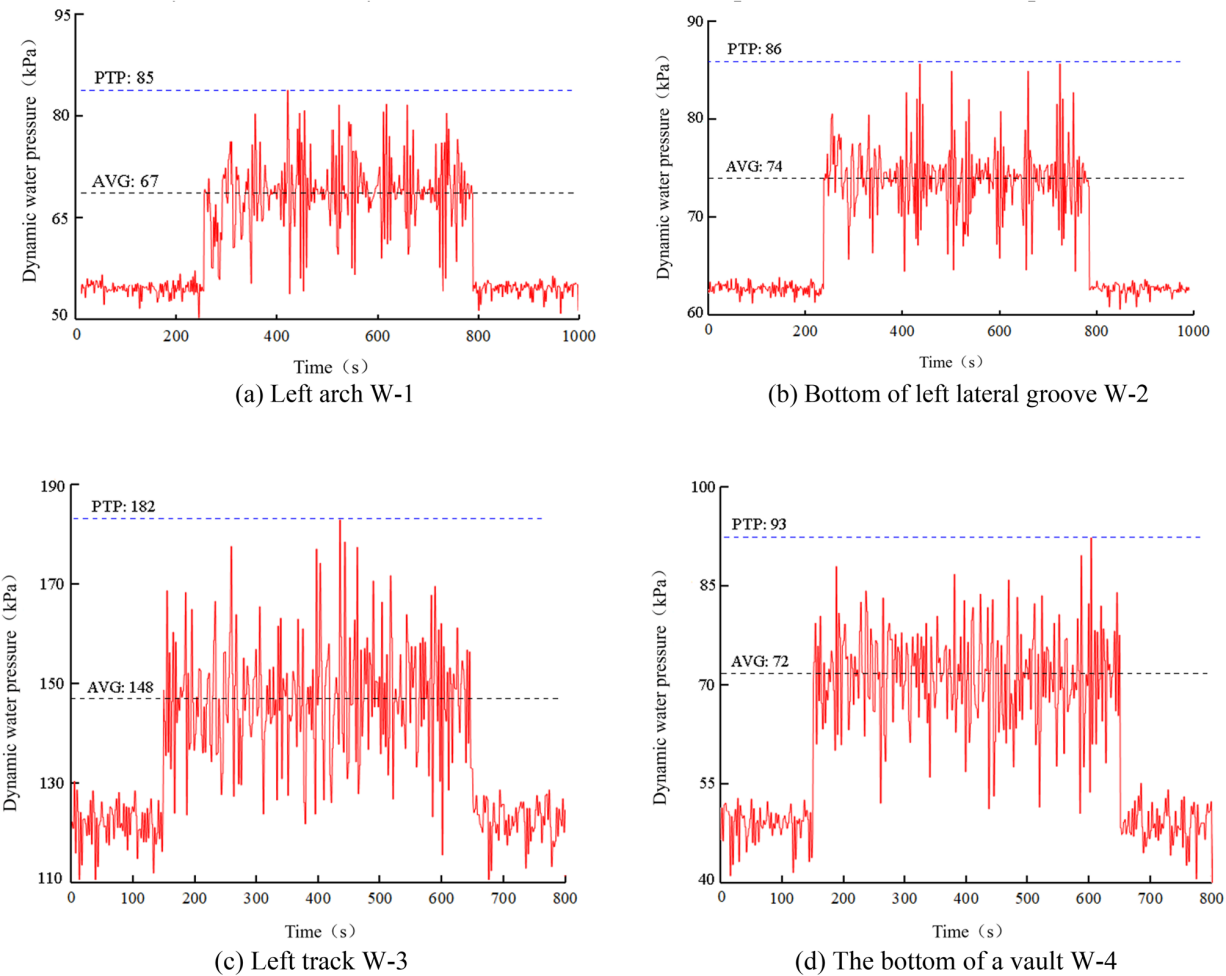
Time history diagram of hydrodynamic pressure on bottom surrounding rock.

As indicated in Fig. 5, a significant increase in water pressure beneath the track and arch bottom occurs under the influence of heavy-haul train loads. Conversely, the increase in water pressure at the left arch foot and bottom of the ditch on the left side was relatively gradual. Over time, the dynamic water pressure damages the rock surrounding the bottom through erosion. The water and soil pressures at the bottom surrounding rock continue to increase over time. The degree of increase in the water and soil pressures varies at different characteristic positions. To explore the correlation between the increase trends of water and soil pressures and the state of the bottom surrounding rock, the water and soil pressures on the bottom surrounding rock were analyzed in stages.

**Figure 6:**
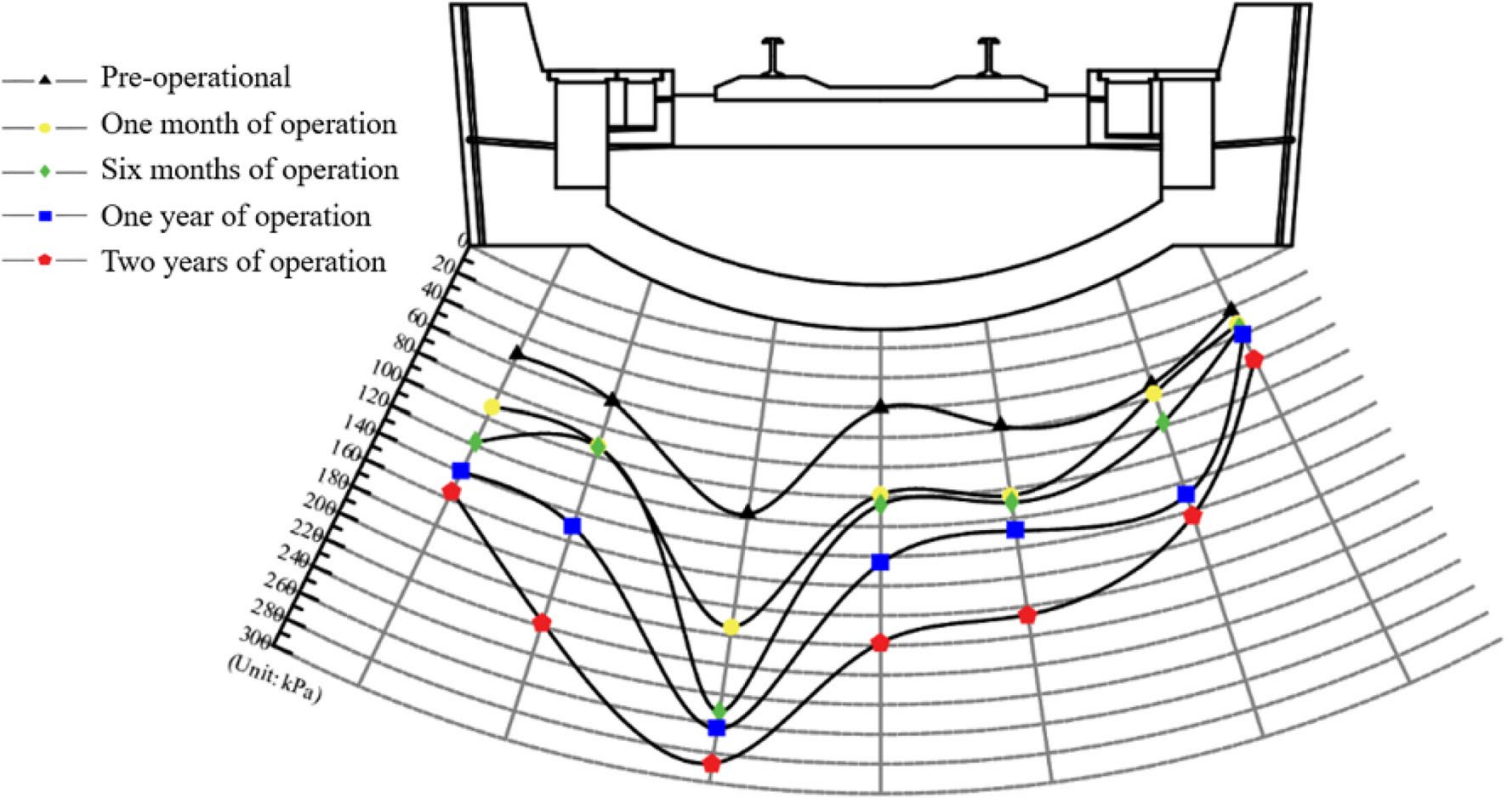
Chart of bedrock surrounding rock water pressure variation.

**Figure 7:**
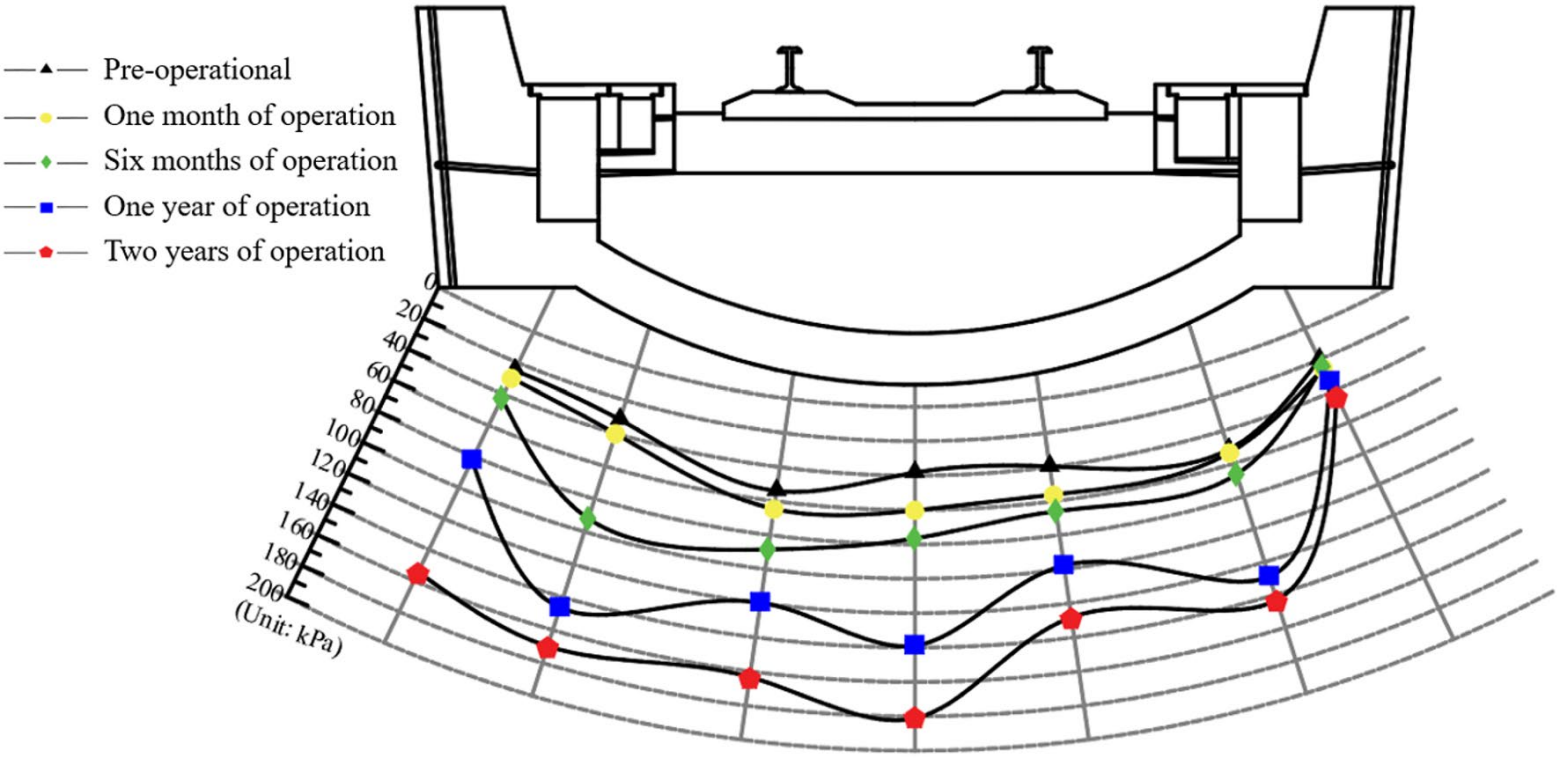
Chart of bedrock surrounding rock soil pressure variation.

As shown in Figs. 6 and 7, the long-term variations in water and soil pressure values on the surface of the bottom surrounding rock are categorized into five characteristic stages based on the service conditions: pre-operational, one month of operation, six months of operation, one year of operation, and two years of operation.

The water and soil pressures on the bottom surface of the tunnel surrounding rock increase continuously over time. After 1 to 2 years of operation, the water pressure at the left rail measurement point increases from 117.35 to 286.86 kPa. The adjacent measurement points (the bottom of the left side ditch and arch) exhibit the highest increase rates in dynamic water pressure, with maximum increments of 217.55 and 198.19 kPa, respectively. The soil pressure on the surface of surrounding rock on the left side was 2–5 times that of the pre-operation state; the increase was significantly higher than that on the right side. The soil pressure at the measurement point of the arch bottom reached a maximum of 181.10 kPa. The bottom surrounding rock continuously deteriorated under the combined effects of groundwater and train loads. Consequently, the soil and water pressures on the rock surrounding the bottom continuously increased over time. This phenomenon tends to destabilize the bottom structure of the tunnel. To explore the impact of these changes on the surrounding rock, further analyses are performed through laboratory experiments and discrete element simulations.

## Experimental study on mechanism of sandstone surrounding rock voiding

The characteristics and formation of voids in sandstone surrounding rock are simulated using indoor experimental models. In the simulation, the bottom rock type, permeability coefficient, tunnel bottom structural form, and high-speed water flow caused by heavy-haul train loads were maintained as controlled variables. The location and degree of defect of the bottom rock, water and soil pressure values of the bottom rock, and degree of soil particle loss were regarded as dependent variables. The void formation process at the bottom surrounding rock of a heavy-haul railway tunnel was deduced by qualitatively analyzing the dependent variables^7,8^.

### Experimental principle

#### Similarity principle

An indoor experiment was conducted using the principle of similarity. The experimental model was constructed based on its similarity in terms of geometry and physics to an actual engineering tunnel structure. In terms of the physical phenomena, the experimental model corresponded proportionally and directionally to the actual tunnel structure at corresponding points. For the dynamic phenomena, the actual underground water flow and water pressure were maintained similar to those in the experimental model. To ensure that the quantitative data obtained from the model experiment could accurately represent the phenomena of the corresponding prototype, achieving geometric, kinematic, and dynamic similarities between the model and prototype was necessary.

The geometric similarity ratio of the experimental model to the prototype was 1:20 based on the principle of similarity. The similarity ratio for volumetric weight is approximated as 1:1. The elastic modulus similarity ratio of surrounding rock to arching concrete plates was 1:20. The bottom structure of the Taihangshan Mountain Tunnel was constructed using plain concrete, indicating that reinforcement was not employed in the tunnel bottom structure model. The similarity constants for other physical quantities are derived based on the π theorem, as summarized in Table 1.

**Table 1:** Similarity constants for indoor tests.

Physical quantity	Similitude parameter
Geometrical dimension, *L* (m)	*C*_*L*_ = 20
Pressure, *P* (kPa)	*C*_*P*_ = 20
Quality density, *C*_*ρ*_ (kg⋅m^−3^)	*C*_*ρ*_ = 1
Elastic modulus, *C*_*Ed*_ (Pa)	*C*_*Ed*_ = 20
Poisson’s ratio, μd	*C*_*μd*_ = 1
Gravitational acceleration, *g* (m⋅s^−2^)	*C*_*g*_ = 1
Dynamic response acceleration, *a* (m⋅s^−2^)	*C*_a_ = 1
Dynamic response pore water pressure, *u* (Pa)	*C*_*u*_ = *C*_*L*_ = 20

#### Bernoulli’s principle

The water flow velocity in the rock surrounding the base was calculated using Bernoulli’s principles. The fixed pipe cross-section was adjusted by varying the height of the water reservoir. Based on the on-site monitoring data, a reasonable range for the instantaneous dynamic water pressure caused by different axle loads of the train was between 0 and 200 kPa. Based on the on-site damage condition, the underground water flow channel was designed as a closed pipe with a diameter of 20 cm and length of 100 cm. The dynamic water flow velocity was calculated using the Darcy–Weisbach formula as follows:

1 $$v = \sqrt {\frac{2Pd}{{\rho fl}}} ,$$

where P is the water pressure in the pipe; *d* is the pipe diameter; *l* is the pipe length; *f* is the pipe friction coefficient; and *ρ* is the flow density. The friction coefficient of the pipe is assumed to be 0.02. Substituting the maximum dynamic water pressure of 200 kPa into the formula yields a maximum dynamic water flow velocity of 63.25 m/s. By converting this velocity using a scale ratio of 1:20, a reasonable range for the dynamic water flow velocity is calculated to be 0–3 m/s. During the experiment, the water flow velocity was controlled by the hanging height of the water reservoir in the experimental setup. The height difference between the liquid level in the water reservoir and inlet is a crucial factor affecting the water flow velocity. Bernoulli’s principle is used to calculate the hanging height of the water reservoir inversely by setting a predetermined flow velocity^9–11^. The detailed calculation is as follows:

2 $$\frac{{p_{1} }}{\rho } + \frac{1}{2}v_{1}^{2} + gh_{1} = \frac{{p_{2} }}{\rho } + \frac{1}{2}v_{2}^{2} + gh_{2} ,$$

where *p* is the fluid pressure; *g* is the gravitational acceleration; *ρ* is the fluid density; and *v* is the fluid velocity. When calculating the flow velocity in English units, the inlet is considered as the reference plane (where *h*_2_ is 0, and *h*_1_ is the target value for the height of the liquid level in the reservoir). Because of the significant difference in diameter between the reservoir and pipeline, area *A*_1_ of the reservoir considerably exceeds area *A*_2_ of the water delivery pipeline. However, *A*_1_*v*_1_ = *A*_2_*v*_2_ (where *v*_2_ is the starting end of water flow) is considerably less than the flow velocity, *v*_2_, at the inlet. Accordingly, *p*_1_ and *v*_1_ can be approximated as 0. Therefore, Eq. (2) can be simplified as follows:

3 $$h_{1} = \frac{{\left( {\frac{{v_{2}^{2} }}{2} + \frac{{p_{2} }}{\rho }} \right)}}{g}.$$

From Eq. (3), *v*_2_ evidently represents the predetermined water flow velocity; constants *ρ* and *g* are known. The desired value of the reservoir liquid level, *h*_1_, can be calculated using only *p*_2_. This was achieved by conducting multiple trial experiments using a pipeline pressure detection instrument. After several rounds of adjustment, the reservoir liquid levels corresponding to flow velocities 1, 2, and 3 m/s were found to be 0.18, 0.34, and 0.6 m, respectively. These values were used to simulate the dynamic water flow resulting from the excitation of heavy-haul trains.

### Experimental design

#### Test materials and devices

For the indoor experiment, rock materials were selected based on the actual conditions in the Taihangshan Mountain Tunnel. Because the actual on-site Class V surrounding rock is predominantly rock, the erosive effects of water on the rock over a short period of time are relatively minimal. Moreover, the necessary water flow conditions for rock erosion are difficult to achieve. Therefore, considering the balance between the timeliness and observability of surrounding rock, the authors decided to focus on the investigation of the surrounding rock soil type. In this indoor experiment, a representative sandstone was selected because of its high observability within a relatively short timeframe. Similar materials are selected for the sandstone surrounding rock; they include bentonite clay, standard sand, talcum powder, and river sand with a mixture ratio of 0.030:0.547:0.035:0.388.

Based on the “Code for Design of Railway Tunnel” (TB10003-2016) and field process experiments for Class V surrounding rock, the range of values for the physical–mechanical parameters of similar materials for indoor rock testing is determined, as listed in Table 2.

**Table 2:** Physical and mechanical parameters of surrounding rock prototypes and similar materials.

Perimeter rock	Severe (kN/m^3^)	Modulus of deformation (MPa)	Poisson’s ratio	Angle of internal friction (°)
Original model	16.8	1000–2000	0.35	20
Similar model	16.8	50–100	0.35	20

**Figure 8:**
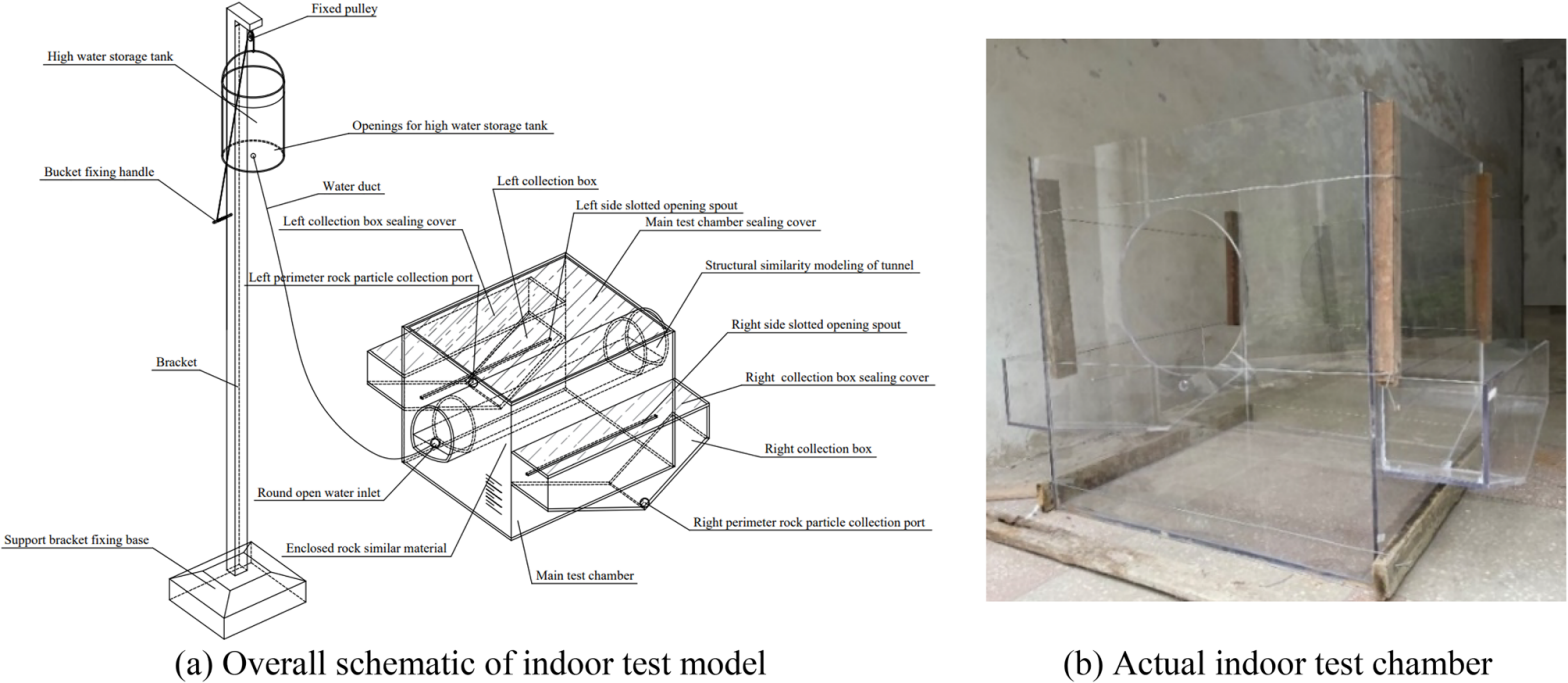
Test setup for surrounding rock spalling.

As shown in Fig. 8, the experimental setup comprises a main test chamber, structural similarity modeling of tunnel, left collection box, right collection box, water duct, high water storage tank, and bracket. The upper part of the main test chamber is sealed. The left and right slotted sides are the opening spouts. The open hole is the water inlet connected to the water duct. The high water storage tank is suspended, and its height is controlled by a pulley fixed to the top of the support frame. The left and right collection boxes share the left and right walls of the main test chamber, respectively. The upper parts of the left and right collection boxes have sealing covers, respectively. The remaining part of the main test chamber is filled with a material similar to that of the surrounding rock.

**Figure 9:**
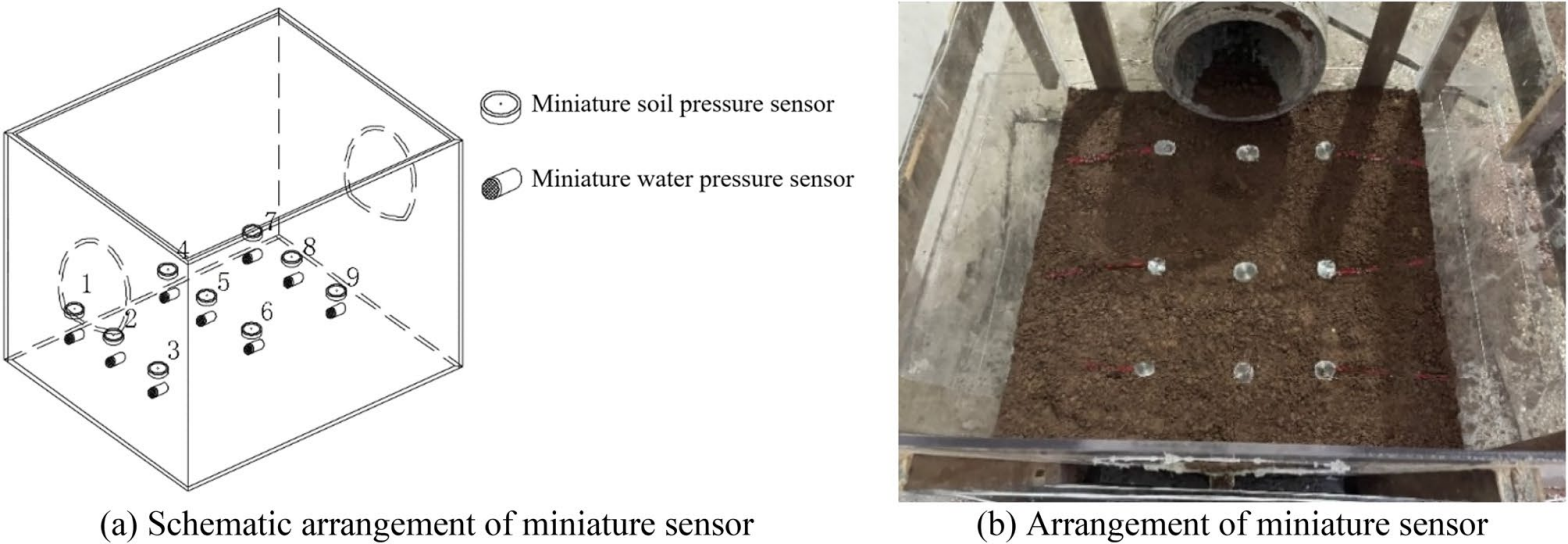
Arrangement of miniature soil and water pressure sensor.

To capture the variations in water and soil pressures during the experiment, miniature water and soil pressure sensors are embedded within a similar material representing the surrounding rock at the bottom of the similar model of the tunnel structure. The sensor parameters are listed in Table 3, and the arrangement of sensors is shown in Fig. 9.

**Table 3:** Parameters of miniature soil and water pressure sensor for indoor testing.

Name	Principle	Measurement range (MPa)	Sizes	Icon
Miniature soil pressure sensor	Resistive strain	0.02 to 10	Caliber: 28 mm Thickness: 10 mm	
Miniature water pressure sensor	Full-bridge resistors	− 0.01 to 5	Caliber: 18 mm Height: 12 mm

As shown in Fig. 9, nine miniature soil pressure sensors and nine miniature water pressure sensors are installed at the bottom of the tunnel model. The miniature water pressure sensor was placed beneath the miniature soil pressure sensor in the same manner as the on-site installation method. These sensors were used to monitor the variations in water and soil pressures during the testing process, reflecting the variations in pressure as the surrounding rock deteriorated and voids developed.

#### Test conditions

The primary focus of this indoor simulation experiment is to study the formation process and characteristics of sandstone surrounding rock voiding under the combined effects of heavy-haul train loads and groundwater. In the indoor test, 1 m/s, 2 m/s and 3 m/s dynamic water flow was used to act on the bottom surrounding rock for a long time, and the soil and water pressure sensor and the shape of the bottom surrounding rock were recorded for the long-term change process. The simulated duration of the experiment was 180 days.

### Test results

**Figure 10:**
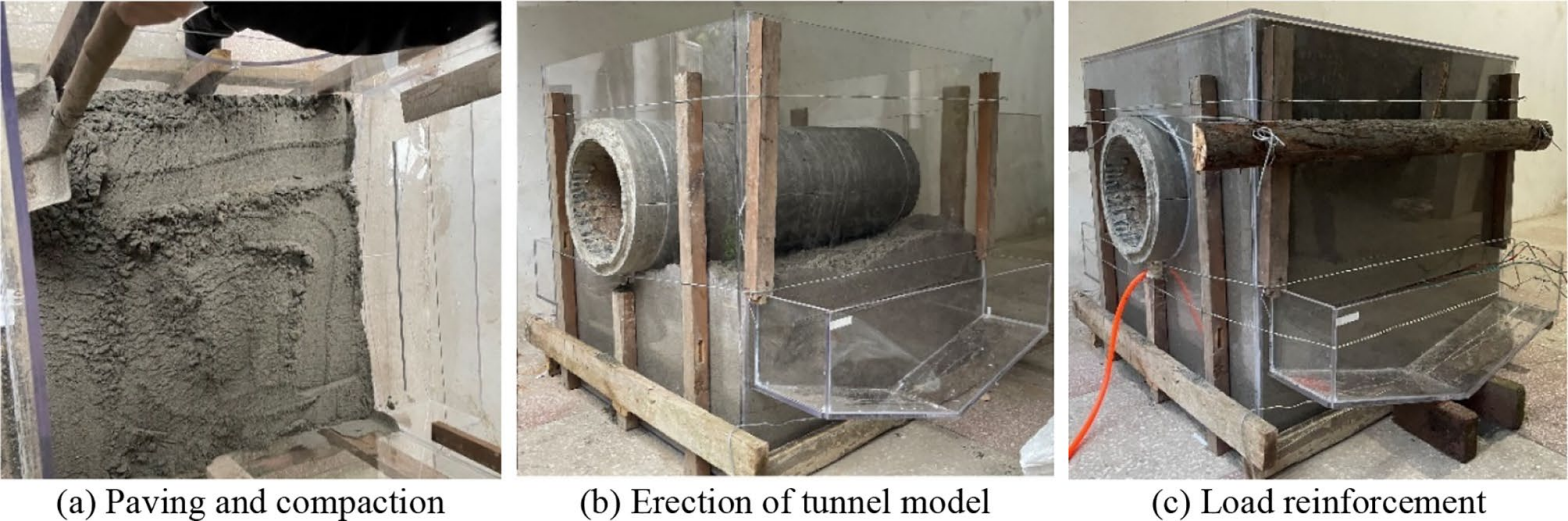
Filling process of sand surrounding rock model.

The sandstone surrounding rock model was constructed using a scaled-down ratio with a mixture of 45 kg of expansive clay, 820 kg of standard sand, 52.5 kg of talcum powder, and 582 kg of river sand. The materials were thoroughly mixed and compacted by layers. The experimental procedure is illustrated in Fig. 10.

**Figure 11:**
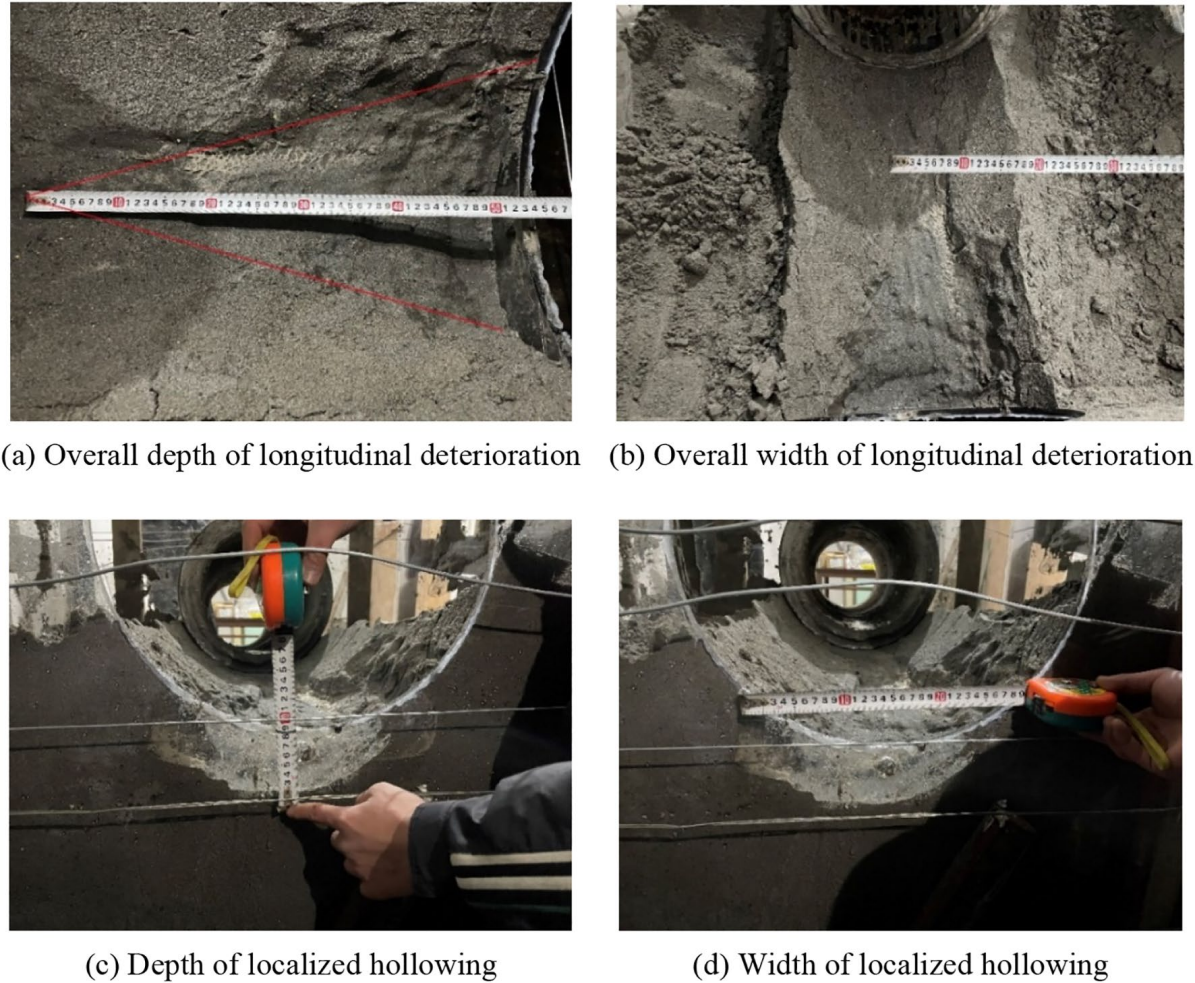
Results of scour erosion of sandy soil perimeter rock bedrock.

Due to scour erosion caused by high-velocity water flow, the sandstone surrounding rock undergoes gradual void formation at its base. As illustrated in Fig. 11, the void develops longitudinally and laterally.

**Figure 12:**
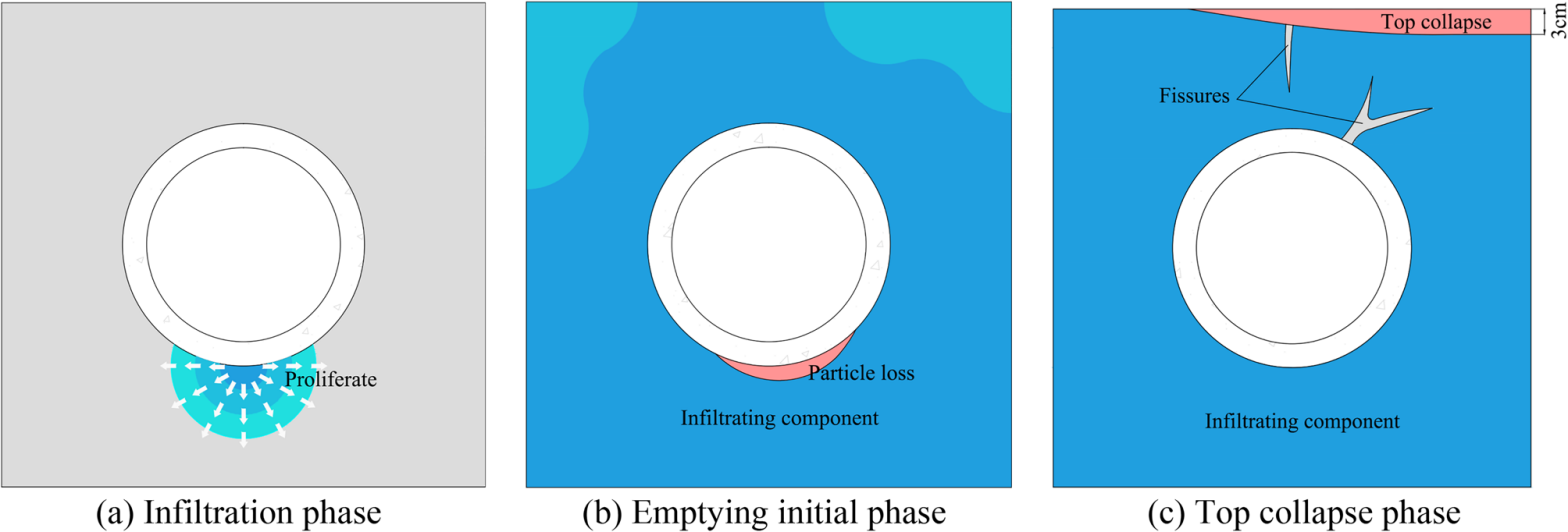
Diagram of experimental process of hollowing of sandy soil surroundings.

Under the scouring action of water flow at a velocity of 3 m/s, the degradation and development of voids at the base of the sandstone surrounding rock are shown in Fig. 12.

As shown in Fig. 12, the degradation process of the sandstone surrounding rock can be broadly divided into three stages: sandstone surrounding rock saturation, particle loss, and localized void formation. In the first stage, the sandstone surrounding rock reaches saturation in a high-water environment, causing a decrease in friction among the rock particles. This leads to a state resembling quicksand, resulting in reduced stability. In the second stage, the dynamic effects of heavy-haul trains generate high-velocity water flow that scours the sandstone surrounding rock. Water flow carries away rock particles, which accumulate at the sides of the testing chamber. In the third stage, the prolonged effects of train loads increase the loss of rock particles, resulting in void formation at the base of the sandstone surrounding rock. This void formation ultimately leads to the overall subsidence and collapse of the top sandstone surrounding rock. The top collapses because its support weakens owing to particle loss and void development at the base.

The longitudinal development pattern of bottom sandstone surrounding rock voiding can be described as follows. The longitudinal influence range of the bottom sandstone surrounding rock voiding formation caused by the water flow induced by heavy-haul trains was approximately 53 cm. As the longitudinal distance from the dynamic action point increased, the influence of the dynamic water flow on the bottom sandstone surrounding rock voiding gradually diminished; the width of the void at the longitudinal void terminal was approximately 9 cm. As for the lateral development pattern of void formation, the largest extent of void formation in the sandstone surrounding rock occurred at the location where the dynamic water flow acted. The depth and width of void formation were 7 and 28 cm, respectively. Upon the completion of the experiment, the rock particles lost during the process accumulated on the storage areas on both sides of the testing chamber. After excluding the excess free water, the weight of the lost particles was determined. The weight of the lost particles in the saturated sandstone surrounding rock subjected to continuous scouring at a water flow velocity of 3 m/s for 1 h was found to be 13.75 kg.

**Figure 13:**
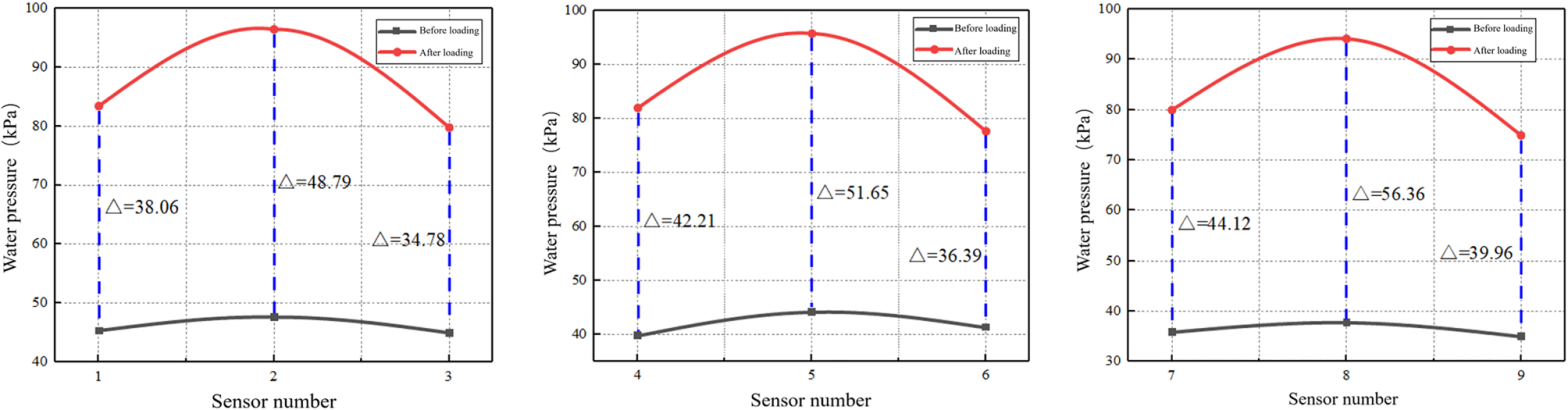
Schematic of instantaneous water pressure.

**Figure 14:**
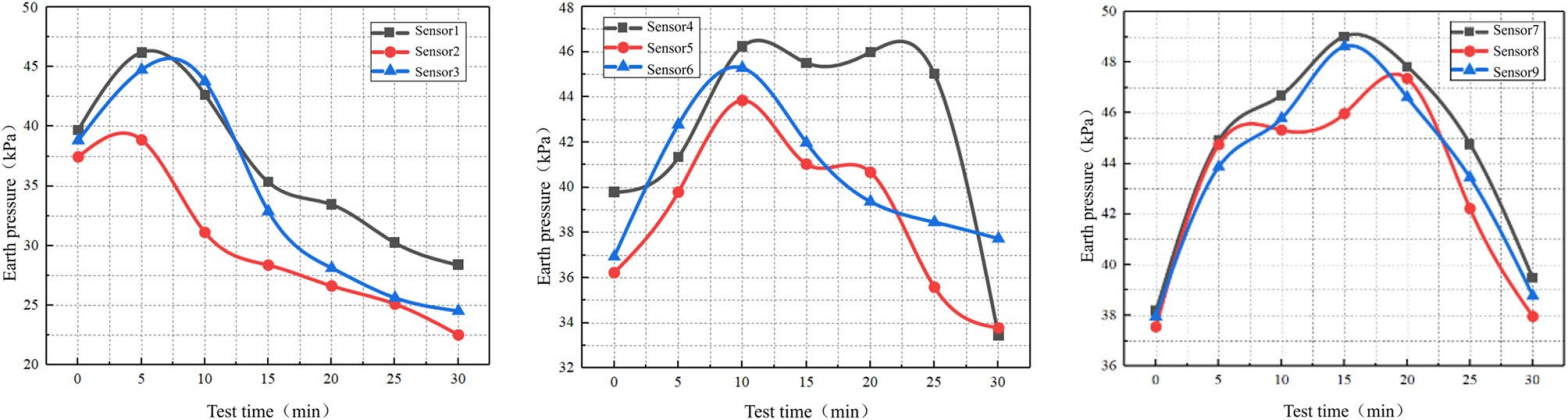
Miniature soil pressure sensor monitoring data.

To gain insight into the sandstone surrounding rock changes during the scouring process, degradation was analyzed by examining the variations in the measurements obtained by the embedded miniature water and soil pressure sensors throughout the experimental process. Detailed information regarding these changes is shown in Figs. 13 and 14.

As shown in Fig. 13, the dynamic water flow exhibits an arc-shaped variation; the maximum water pressure is located at the center of flow. As the lateral and longitudinal distances increased, the water pressure gradually decreased. In the same hydrogeological environment, a high water pressure magnitude has a significant impact on sandstone surrounding rock scouring. High water pressure results in high initial velocity for rock particle movement under dynamic water flow, leading to long migration distances and large void formation areas. This pattern of lateral and longitudinal water pressure variations corresponds to the extent of void formation at the bottom sandstone surrounding rock.

As shown in Fig. 14, as the degradation of sandstone surrounding rock caused by water flow progresses, the sensor located at the far end away from the direct action of the dynamic water flow is least affected. Throughout the experimental process, the trends and magnitudes of the changes were similar for all sensors. The maximum increase in readings owing to the compression experienced by the sensor at the far end was 28%. However, as the loss of rock particles increased and void formation occurred, the reduced constraint in all directions led to a gradual decrease in soil pressure measured by the sensors. This indicates that void formation has a certain impact on the stress distribution on the sandstone surrounding rock and the distribution of water and soil pressures.

**Figure 15:**
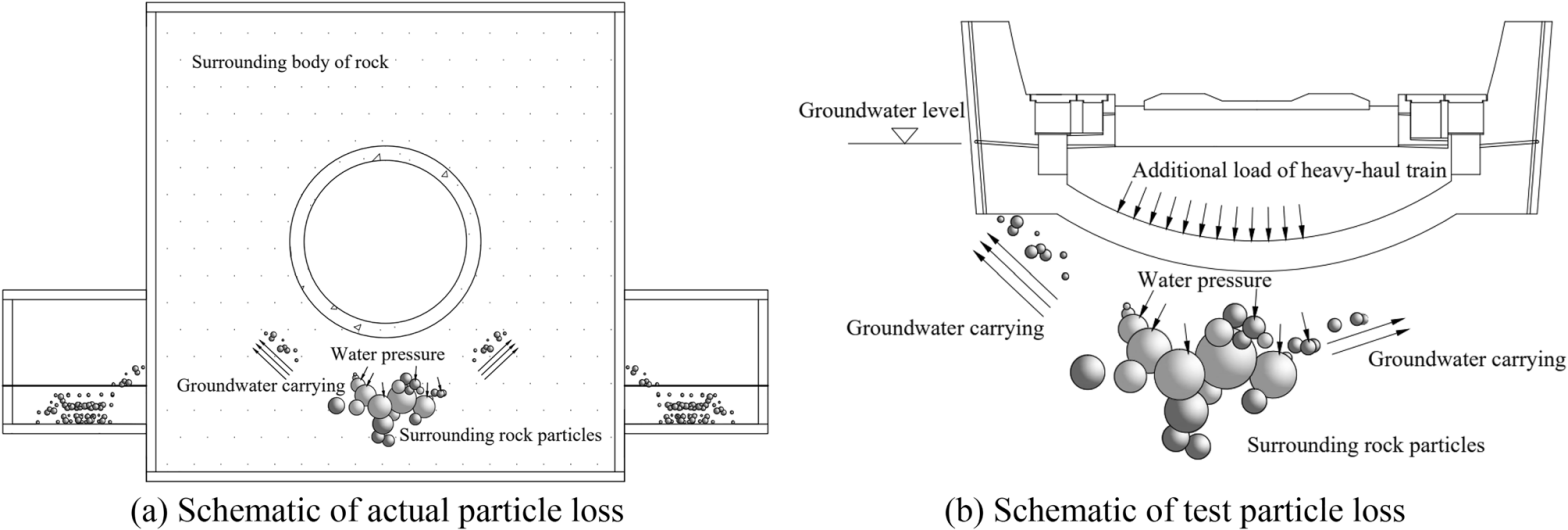
Schematic of bottom perimeter rock affected by groundwater scouring.

The results of the indoor experiment revealed that in the presence existing defects in the tunnel floor and bottom sandstone surrounding rock, the dynamic loads induced by the heavy-haul train action generated extremely high water pressure. Prolonged exposure to a dynamic water flow causes the removal of rock particles, resulting in void formation. Furthermore, this void formation exacerbates the defects in the bottom sandstone surrounding rock. A detailed schematic of this process is presented in Fig. 15. The observed changes in water and soil pressures in the bottom sandstone surrounding rock of the Taihangshan Mountain Tunnel lead to the conclusion that the groundwater scouring flow beneath the bottom sandstone surrounding rock is intensified by the combined effects of time and heavy-haul train loads. Consequently, the initially loose rock particles are dislodged, as shown in Fig. 15b. The repeated action of water and load over time leads to the formation and further development of voids in the tunnel floor structure. Using the discrete element method, numerical simulation analysis was conducted to study the particle loss and analyze the mechanism of void formation in the bottom sandstone surrounding rock of heavy-haul railway tunnels.

## Simulation and discrete element analysis of basement perimeter rock devolatilization

A model was established using the PFC discrete element method combined with remote field measurements and indoor experimental data. The groundwater pressure induced by train vibrations was converted into erosion at varying flow velocities to simulate the scouring and erosion of sandstone surrounding rock. The discrete element model is characterized by discontinuity and nonlinearity, effectively simulating the damage and failure mechanisms of rock microstructures. It authentically and visually represents the manner in which rock particles detach and are lost under the action of groundwater scouring. It provides insight into the form and extent of void formation under different rock conditions and water flow velocities. Accordingly, the discrete element model is used in the simulation to study the mechanism of void formation in the sandstone surrounding rock as well as the range and degree of degradation under the action of scouring.

### Calibration of perimeter rock parameters

Numerical simulation experiments were conducted to calibrate the model and parameters of sandy rock. The model has four sides: the upper and lower sides are primarily controlled by velocity to simulate loading, and the other two sides simulate rock pressure. The PFC discrete element software was employed to analyze the degradation mechanism and extent of void formation in the bottom sandstone surrounding rock. Prior to the analysis, determining the microscale parameters of the sandstone surrounding rock is crucial to ensure the accuracy of the simulation^12–14^. In this study, the numerical simulation test of indoor triaxial test is simulated by PFC biaxial compression unit test. By conducting a large number of PFC biaxial compression numerical tests, the PFC fine-scale parameters matching the macroscopic parameters of the material triaxial test can be obtained.

### Tunnel base dynamic water modeling

**Figure 16:**
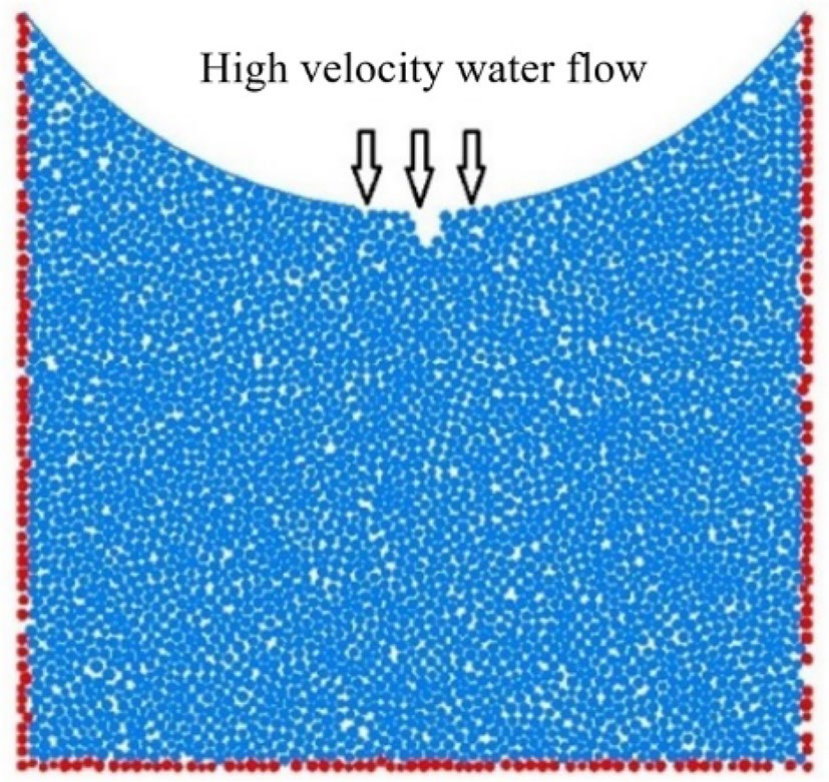
Schematic of dynamic water flow applied to granular flow model.

In this study, dynamic water pressure values are determined based on previous field-monitoring data from the Taihangshan Mountain Tunnel. By analyzing the time history curve of the measured arch water pressure on-site, the pore water pressure at various monitoring points was observed to increase over time in the range 0–200 kPa. Based on these data, a water flow range of 0–3 m/s is selected. The particle flow model is shown in Fig. 16.

**Figure 17:**
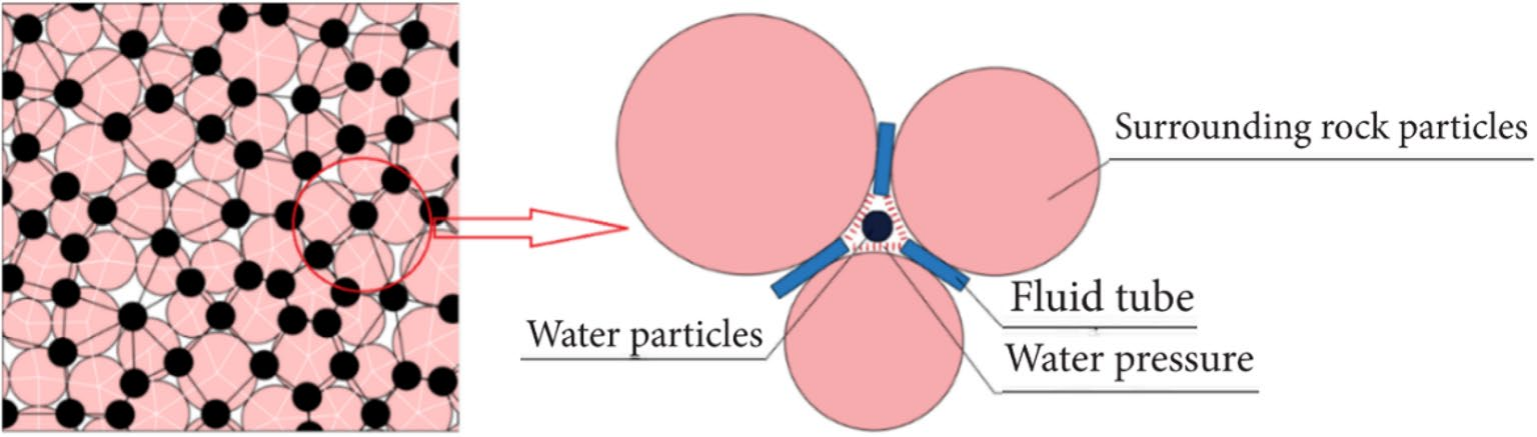
Flow network model.

As shown in Fig. 16, the red spheres surrounding the model represent fixed and impermeable boundaries. The top was not constrained to simulate a water channel for particle loss. Some particles near the bottom of the arch were removed to simulate the initial defects in the sandstone surrounding rock. A boundary with an adjustable velocity was applied to simulate the high-pressure pore water flow. In high-pressure flow fields, a flow network model is generally employed to simulate fluid–solid coupling. After generating the sandstone surrounding rock model, water particles were introduced at the contact points of rock particles. These water particles were then interconnected to form flow channels, ultimately creating a comprehensive network of flow channels. During the calculation process, water pressure was continually updated and applied to the surrounding rock particles. This reflects the movement and loss of rock particles under the influence of water flow^15–17^. The flow network model is shown in Fig. 17, and the microscale parameters of the fluid grid are listed in Table 4. The experimental conditions are listed in Table 5.

**Table 4:** Microscopic parameters of fluid grid.

Fluid density (kg/m^3^)	Water velocity (m/s)	Coefficient of viscosity (Pa s)	Permeability coefficient (cm/s)
1000	0–3	1.0 × 10^−3^	4.0 × 10^−9^–1.0 × 10^−4^

**Table 5:** DEM calculation conditions.

Working condition number	Water velocity (m/s)	Simulation time (days)
1	1	0–360
2	2	
3	3	

### Simulation of water flow channel formation

**Figure 18:**
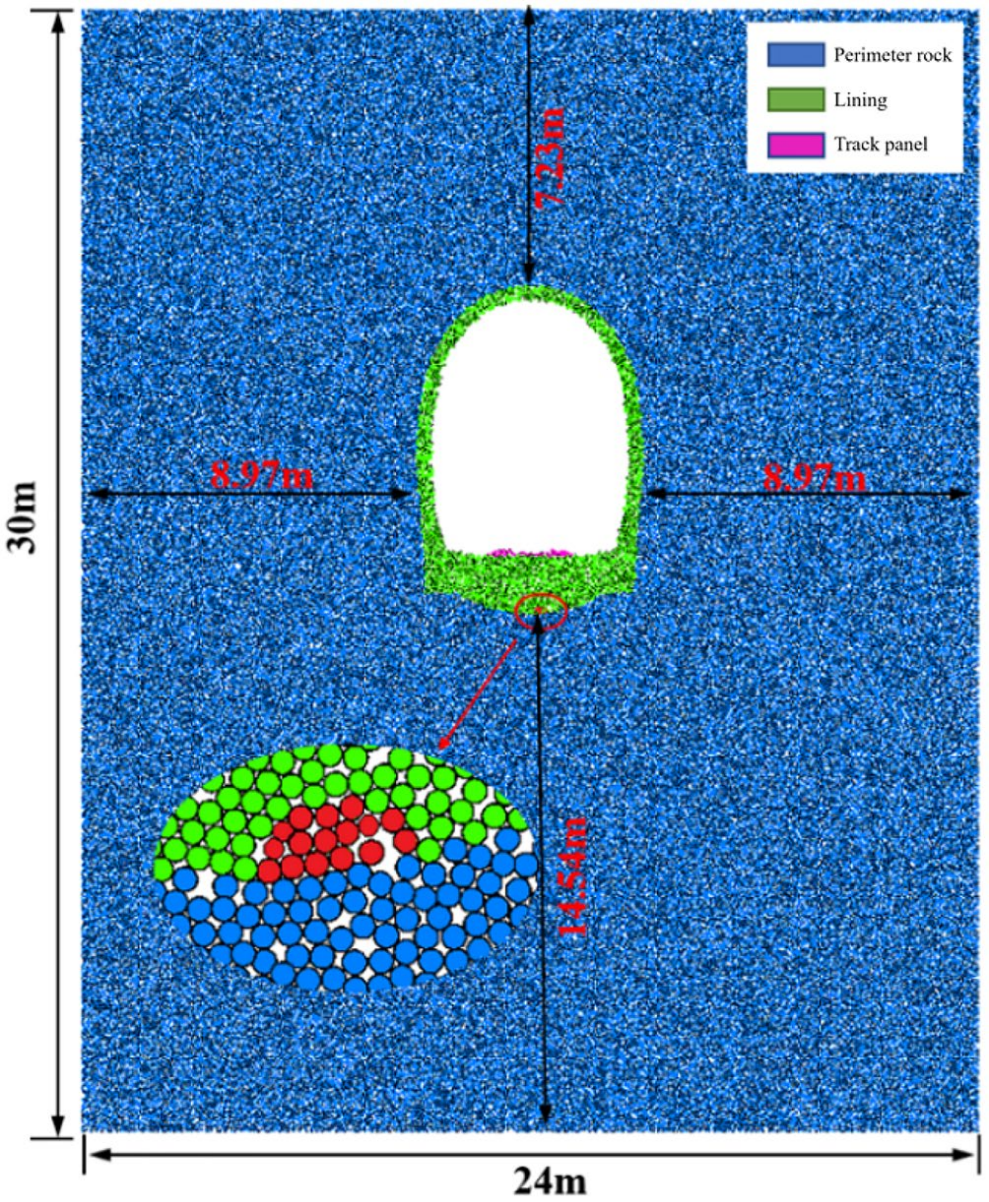
Overall model of base containing slag.

Based on field investigations, achieving a seamless fit between the sandstone surrounding rock and arch structure during the construction phase is evidently challenging. The locations of voids or loose materials in the sandstone surrounding rock become pathways for the loss of rock particles under the combined effects of train loads and water flow. This implies that the occurrence of void formation solely because of scouring is improbable. The existence of initial damage and space at the bottom is a prerequisite for void formation. Considering the dimensions of the Taihangshan Mountain Tunnel section as reference, an integrated model was developed to account for the presence of voids or loose materials in the sandstone surrounding rock. The dimensions of the integrated model are 30 m × 24 m. This includes a layer of void particles at the bottom of the arch structure, measuring 20 cm in width and 15 cm in height, as shown in Fig. 18.

Based on the current “Code for Design of Railway Tunnel” (TB10003-2016) and the calibration results, the microscale parameters of sandstone surrounding rock particles were selected. The material parameters are summarized in Tables 6 and 7.

**Table 6:** Macroscopic mechanical parameters of materials.

Material	Density (kg/m^3^)	Deformation modulus(kg/m^3^)	Pisson’s ratio	Angle of internal friction (°)	Cohesive force (kPa)
Lining structure	2600	15.4	0.2	30	0.8
Filling of superelevation arches	2500	12.5	0.2	18	0.4
Class V perimeter rock	2150	2	0.35	20	0

**Table 7:** Microscopic parameters of tunnel structure.

Microscopic parameters	Lining structure	Filling of superelevation arches
Particle normal stiffness (N/m)	3.0 × 10^9^	1.4 × 10^9^
Particle tangential rigidity (N/m)	3.0 × 10^9^	1.4 × 10^9^
Parallel bonding normal stiffness (N/m)	1.2 × 10^10^	9.1 × 10^9^
Parallel bonding tangential stiffness (N/m)	1.2 × 10^10^	9.1 × 10^9^
Parallel bonding normal strength (N)	2.1 × 10^6^	1.2 × 10^6^
Parallel bonding tangential strength (N)	2.1 × 10^6^	1.2 × 10^6^

**Figure 19:**
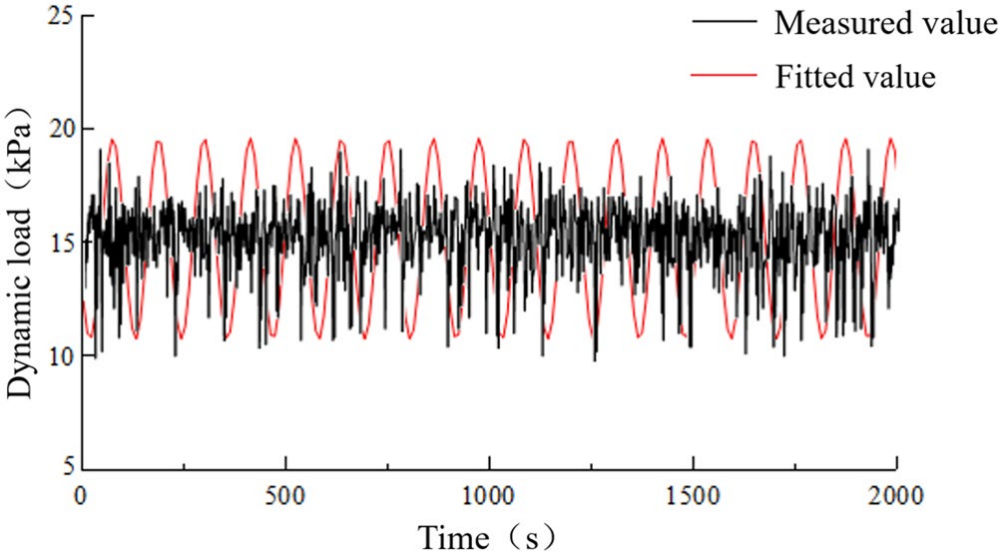
Load-time history curve.

Based on data monitoring data, the on-site foundation dynamic loads were simplified as a sinusoidal function applied to the position of the track slab. The track slab was considered as a rigid wall. Acceleration was applied to the wall, indirectly transferring velocity to the particles of the underlying surrounding rock. This process also realistically replicates the transmission of force from an actual train load. The measured dynamic load time history curve and fitted sinusoidal function are shown in Fig. 19.

**Figure 20:**
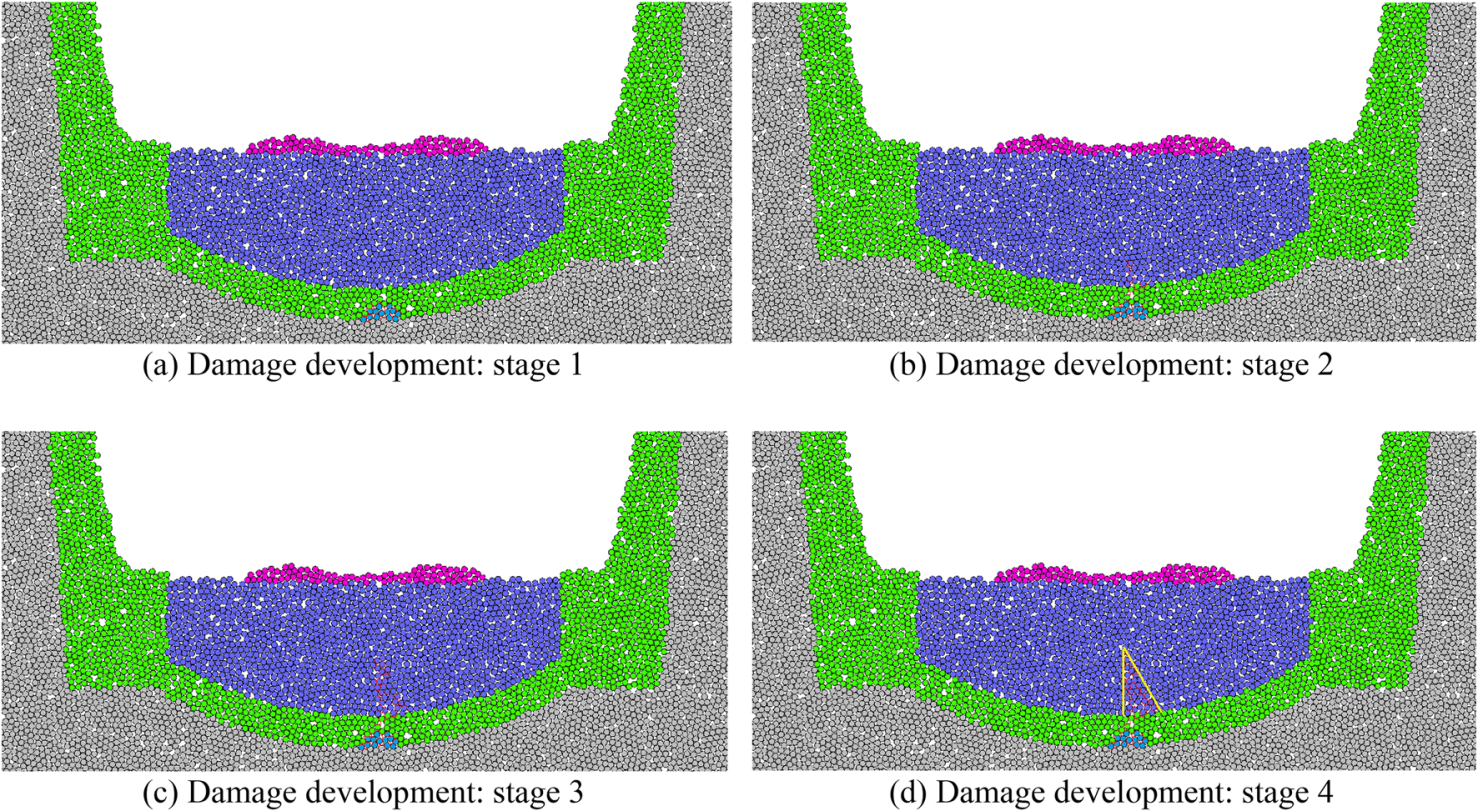
Deterioration process of defective inverted arch.

When voids exist between the lining structure and underlying surrounding rock, the desired thickness cannot be achieved by the inverted arch structure. Under the influence of train dynamic loads, the uneven positions of voids at the bottom surface of the inverted arch can lead to stress concentration. This results in uneven force distribution and insufficient bearing capacity of the lining structure. Damage to the inverted arch structure owing to cyclic train loading is shown in Fig. 20.

As shown in Fig. 20, material differences exist between the particles of surrounding rock and those in the inverted arch structure. The particles adhere to each other because of compressive forces, resulting in relatively weak interactions among the particles. During the initial stages of heavy-haul train application, a significant number of deteriorating cracks form at the void locations. Because the voids occupy a portion of the inverted-arch thickness, this area becomes relatively weak. Under the influence of heavy-haul train loads, significant tensile stresses develop at these locations. Consequently, the cracks gradually propagate from the stress concentration points toward the filling material of the inverted arch.

### PFC2D modeling of perimeter rock voiding

**Figure 21:**
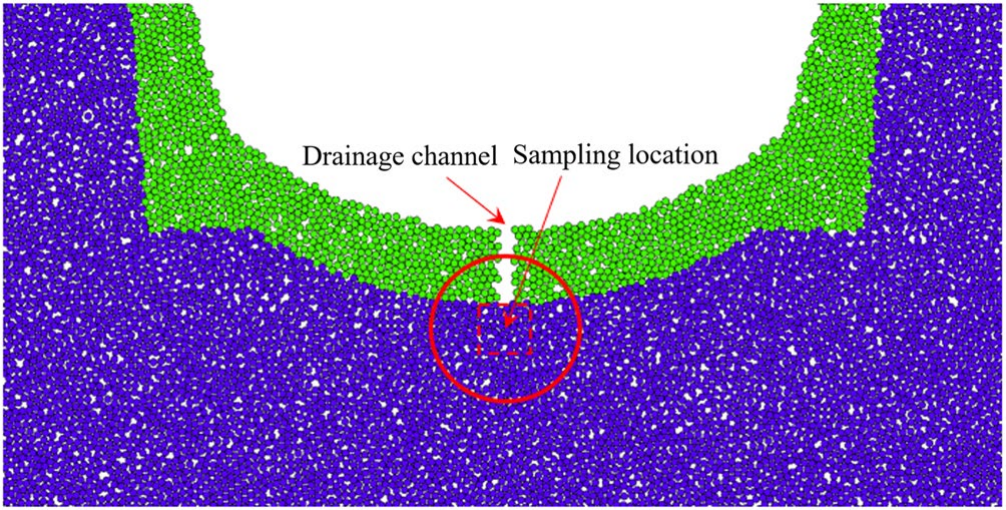
Macroscopic position of particle flow in weak surrounding rock model.

**Figure 22:**
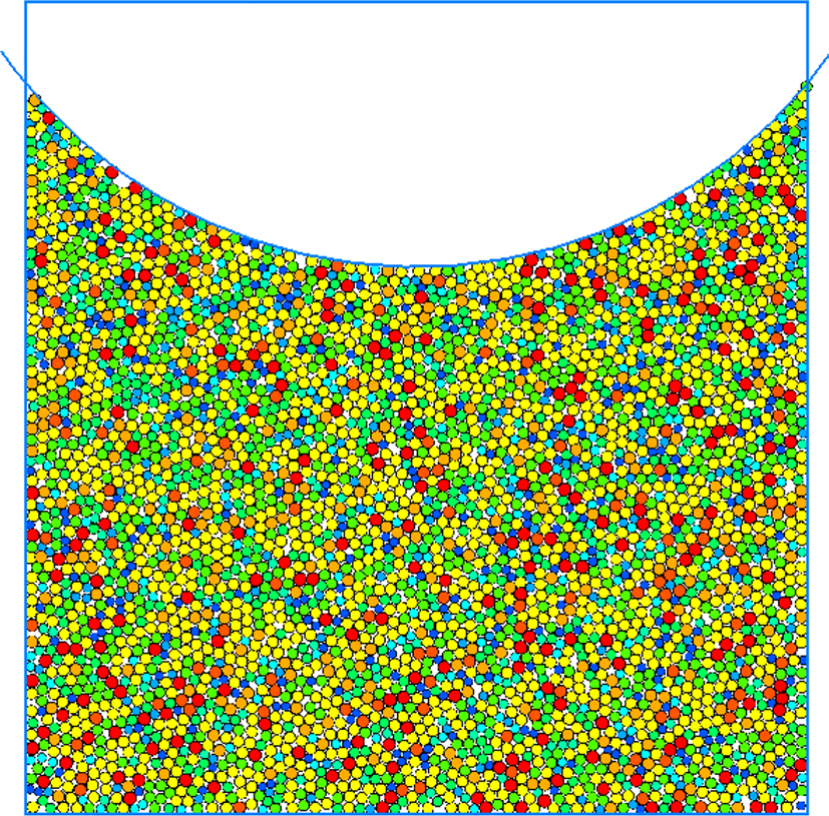
Granular flow in soft weak rock model.

The analysis of measured results indicates that the detachment of surrounding rock in the heavy-haul railway tunnel mainly occurs at the track and central line positions. This phenomenon is primarily attributed to the deterioration of the surrounding rock caused by the interaction between train loads and groundwater. Considering the computational efficiency of the PFC2D particle flow software, creating an overly large model is not advisable. Accordingly, the current PFC2D discrete element model was simplified. It is focused on analyzing the local sandstone surrounding rock at the contact position between the tunnel’s inverted arch structure and surrounding rock, as shown in Figs. 21 and 22.

In the PFC2D model, the materials interact with rigid spheres. In the current study, the particle material of the sandstone surrounding rock model is linear. The model comprises two parts: inverted arch and surrounding rock. Specimen loading was simulated by controlling the velocity of the inverted arch wall. For computational convenience, the irregular rock particles in the surrounding rock were idealized as regular circular particles in this model.

### Surrounding rock analysis result

#### Perimeter rock degradation pattern

**Figure 23:**
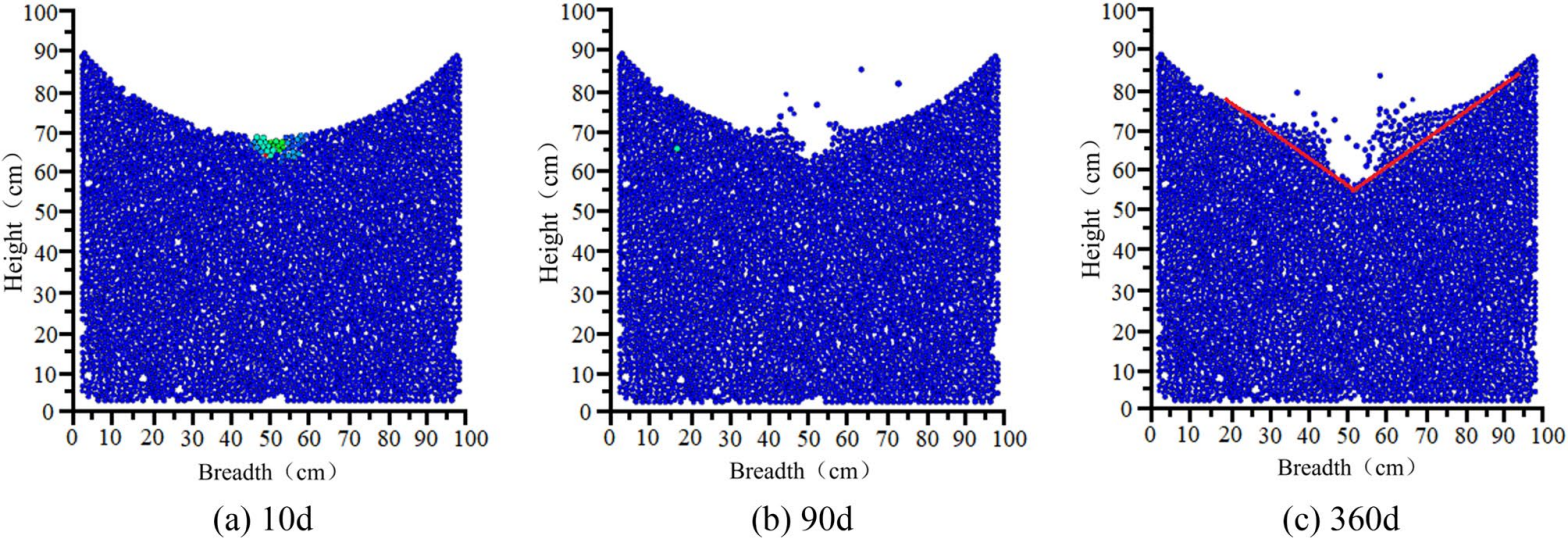
Void development in sandstone surrounding rock.

The variations in the degradation and detachment of sandstone surrounding rock are illustrated in Fig. 23.

The variations in sandstone surrounding rock degradation and detachment during different stages indicate that when suspended particles are excluded, the overall pattern approximately forms a “V” shape distribution. Over time, the depth of the “V” shape gradually increases. This phenomenon is attributed to the fact that the bottom structure of the model has an inverted arch shape that naturally comes into contact with the surrounding rock, thereby forming a natural arched surface. This results in the formation of a natural slope on both sides of the water outlet. Additionally, owing to the loose nature of sandy soil, the rock particles on the slope surface gradually slide and erode as the groove deepens. Detailed information based on the statistical analysis of the degree of detachment during each operational stage is summarized in Table 8.

**Table 8:** The Sandstone surrounding rock caving and deterioration development table.

Operating hours (day)	Number of particles lost from the surrounding rock	Shedding width (cm)	Shedding depth (cm)
10	0	0	0
30	3	12	6
60	15	13	8
90	27	15	8
180	42	32	10
360	78	43	15

#### Peripheral rock particle contact chain

**Figure 24:**
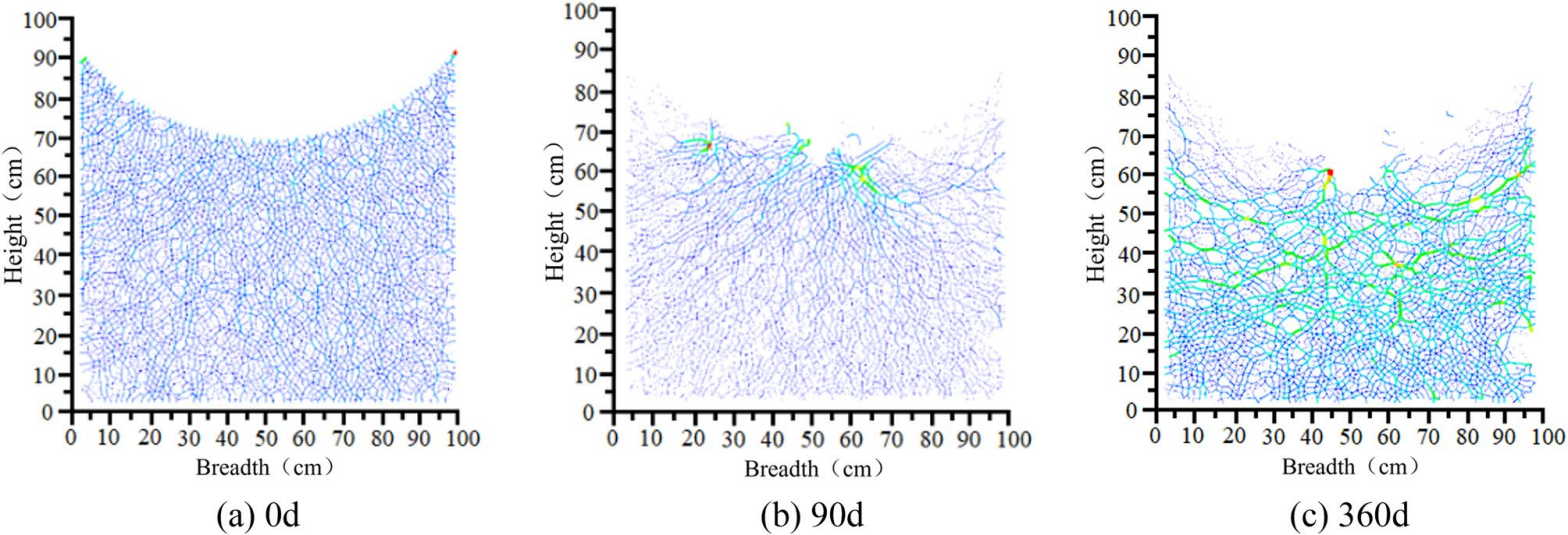
Distribution of particle contact chains in sandstone surrounding rock.

In addition to reflecting the degradation of the sandstone surrounding rock visually through particle loss, the assessment can be based on the contact state among rock particles. The movements and changes in the particles are reflected by their interactions at contact points. The mechanical relationships among the particles can also be analyzed based on contact chains. The changing state of particle chains at selected characteristic nodes under the influence of dynamic water flow is shown in Fig. 24.

As shown in Fig. 24, under the no-water condition prior to operation, the distribution of sandstone surrounding rock particle contact chains is uniform and has no significant defects. A few particles experience considerable forces owing to wall compression only at the apex of the arch. After 60 days of operation, some particles began to erode, and the erosion gaps in the particle contact chains further expanded. However, the extent of degradation did not increase considerably. Notably, the degradation in the width direction of the sandstone surrounding rock is more distinct. This is a significant factor leading to the “crescent-shaped” detachment pattern observed in the laboratory tests of sandstone surrounding rock. After 180 days of operation, the vertical pressure on the sidewall particles at the arch’s sloping base increases as the lateral constraint decreases. This leads to the weakening and even complete failure of particle contacts above the water flow surface.

## Deterioration attenuation law of bottom surrounding rock

### Influence of perimeter rock wastage rate on deterioration

**Figure 25:**
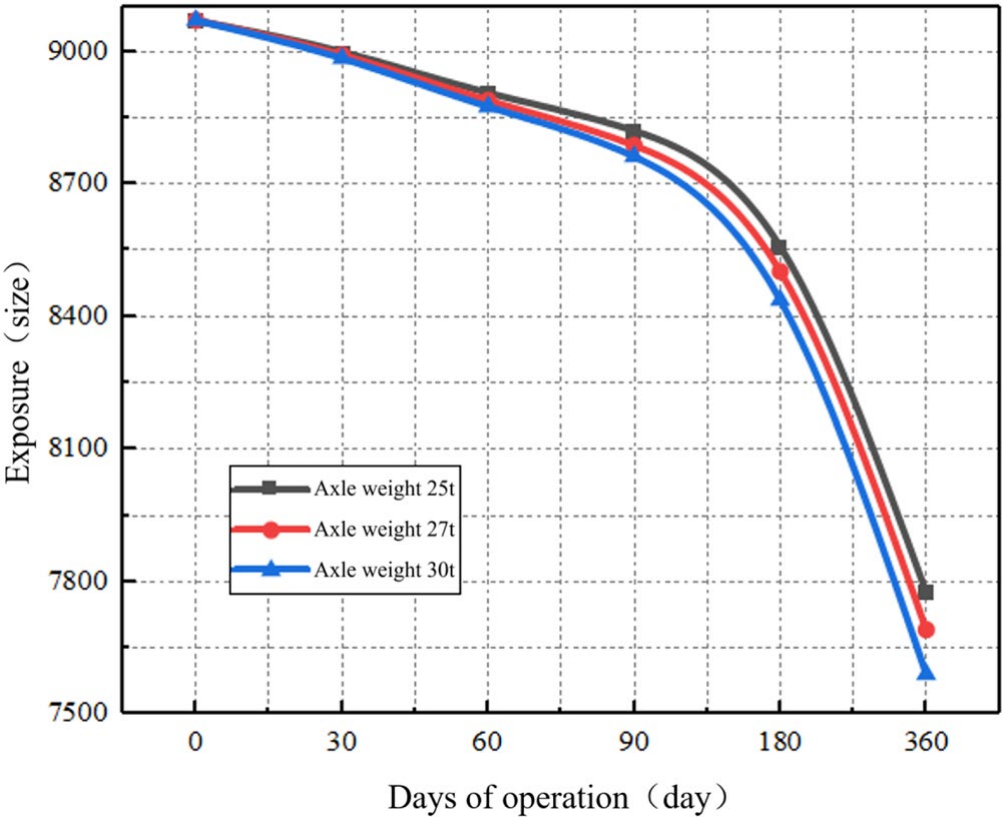
Contact failure development diagram of each operating node under different axle load conditions.

The loss of sandstone surrounding rock particles is a process of damage accumulation. Quantitative analysis of the wastage rate was conducted by assessing the number of failures in the indirect contact among rock particles. The analysis aids in understanding the relationship between wastage rate and operational time based on the detachment results of sandstone surrounding rock during different operational stages. The relationship between the number of contact nodes and operational time under different axle load conditions is shown in Fig. 25. The Development of wastage rate of each operation node in Table 9.

**Table 9:** Development of wastage rate of each operation node.

Axle Weight (t)	0 day	Wastage rate (%)	30 days	Wastage rate (%)	60 days	Wastage rate (%)	90 days	Wastage rate (%)	180 days	Wastage rate (%)	360 days	Total (%)
25	9070	0.78	8999	1.03	8906	0.97	8820	2.97	8558	9.14	7776	14.27
27	9070	0.86	8992	1.15	8889	1.15	8787	3.24	8502	9.53	7692	15.19
30	9070	0.95	8984	1.21	8875	1.27	8762	3.71	8437	10.03	7591	16.31

**Figure 26:**
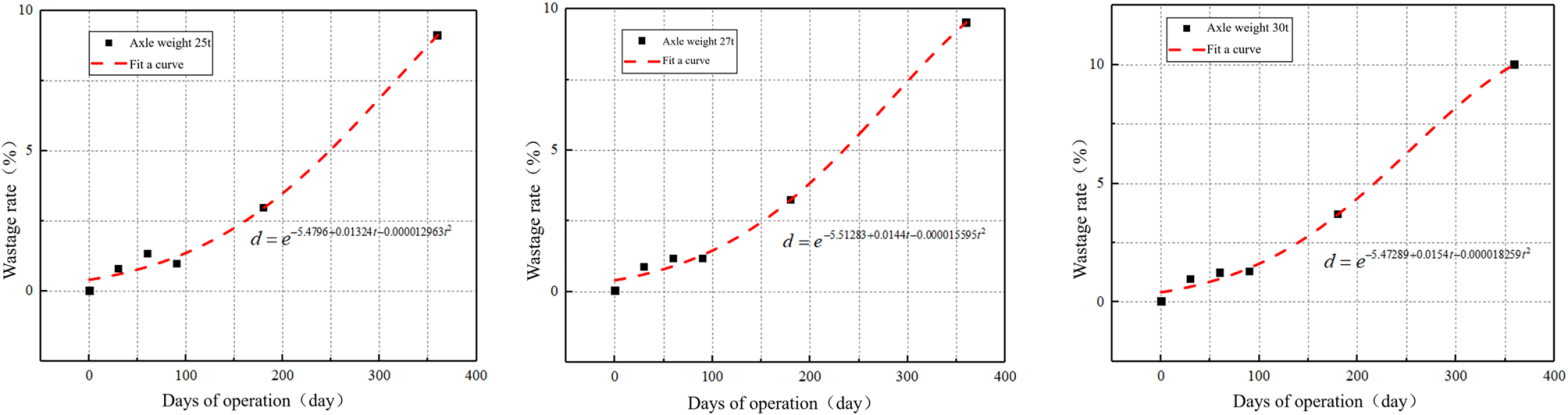
Axle load operating time–wastage rate diagram.

As shown in Fig. 25, the degradation rate of sandstone surrounding rock is relatively gradual during the first 180 days; thereafter, the degradation rate increases. For the sandstone surrounding rock with the same particle quantity and size ratio, the initial contact quantity remains the same. By fitting the wastage rate at various characteristic operational time points, the relationship between the wastage rate of the sandstone surrounding rock and operational time under different axle load conditions can be determined, as shown in Fig. 26.

**Figure 27:**
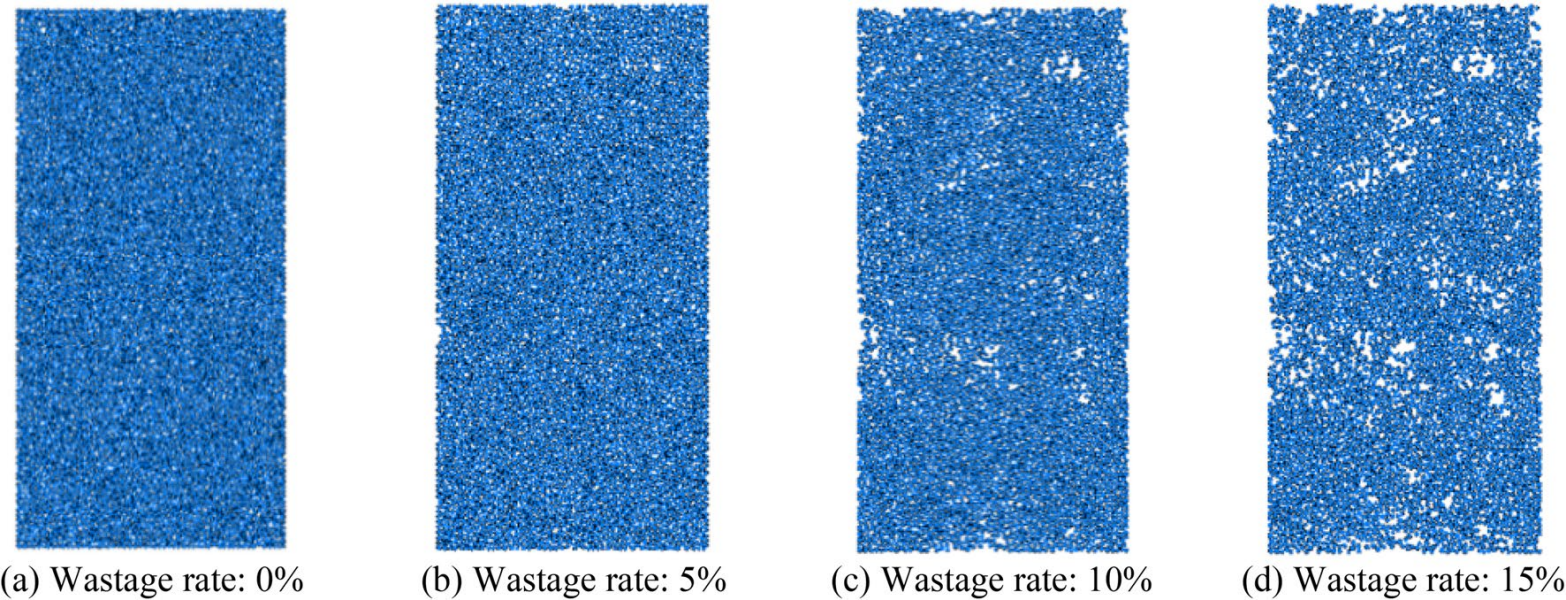
Loss models.

Based on Fig. 26, the fitted equation for the axle load operating time–wastage rate of surrounding rock is as follows:

4 $$d = e^{{\left( { - 5.47289 - 0.03994e^{{ - \frac{{\left( {Z - 27} \right)^{2} }}{2.26585}}} } \right) + e^{{ - 8.0221 + 0.24597Z - 0.00392Z^{2} }} t - \left( { - 0.00002269 + 0.00049686e^{{ - \frac{Z}{6.3552}}} } \right)t^{2} }} ,$$

where *d* is the wastage rate; *Z* is the axle weight; and *t* is the operating time. The loss of sandstone surrounding rock particles is a critical factor directly affecting the integrity of the surrounding rock. To analyze the condition of the sandstone surrounding rock under different wastage rates, three loss models were established based on wastage rates of 5%, 10%, and 15%. Considering the Class V surrounding rock as an example, the attenuation of the surrounding rock strength under different wastage rates is analyzed. The models are shown in Fig. 27.

**Figure 28:**
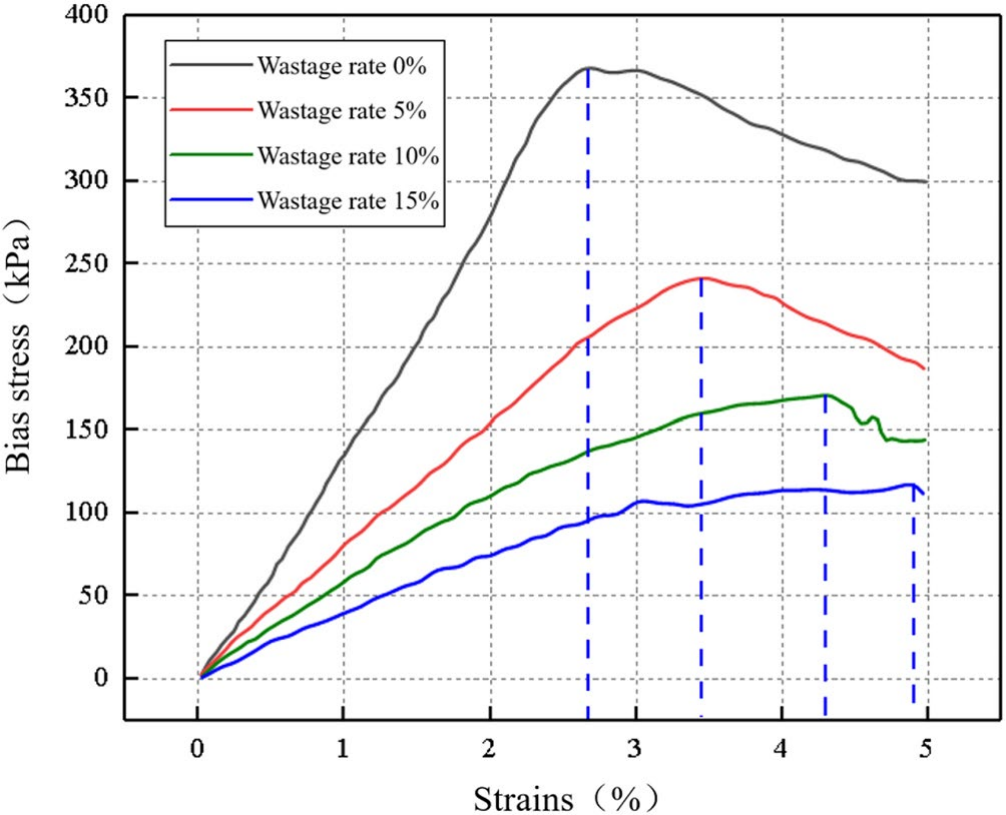
Stress–strain diagram under different wastage rates.

As shown in Fig. 28, as the wastage rate increases, the peak of the stress–strain curve decreases, and the failure strain of the surrounding rock increases. This indicates that the overall integrity of sand surrounding rock decreases after the loss of rock particles, thereby altering the initial force transmission paths among the particles. This leads to the loosening of surrounding rock particles around the loss voids. This breaks the isotropic characteristics of the internal structure of sandstone surrounding rock. The change trend of the stress–strain curve indicates that parameters, such as sandstone surrounding rock failure strength, deformation degree, and elastic modulus, attenuate.

### Elastic model decay laws

**Figure 29:**
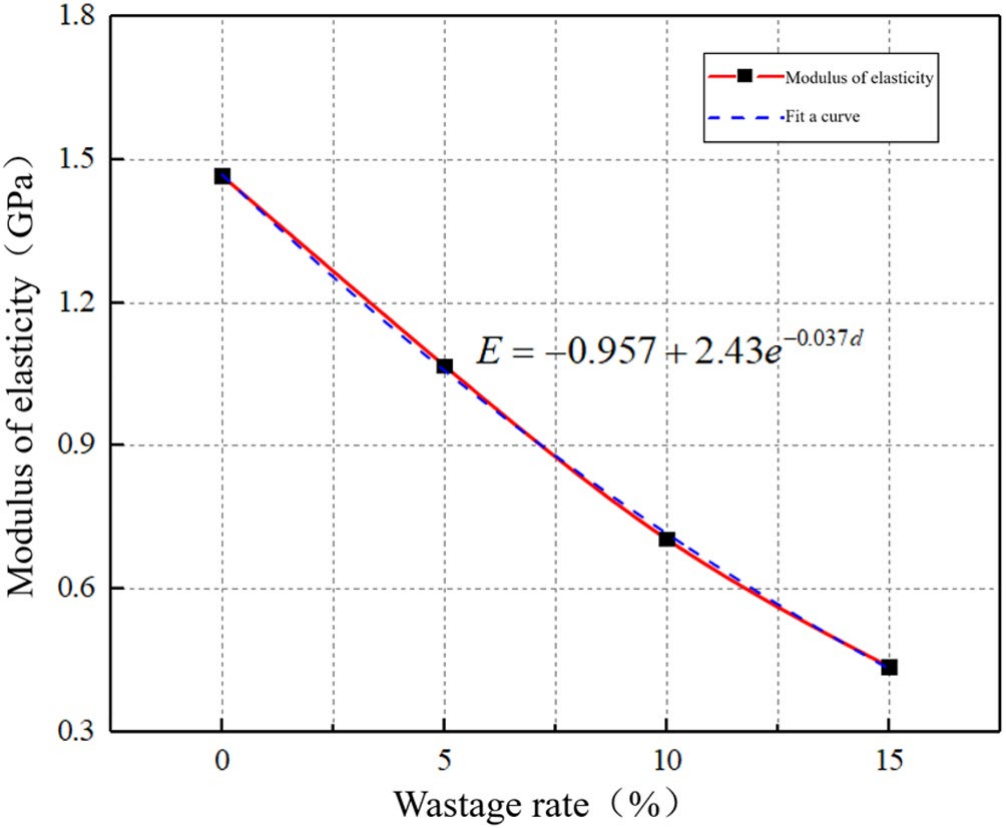
Elastic modulus attenuation curve.

Based on the stress–strain curves of surrounding rock under different wastage rates, an increase in wastage rate evidently leads to a densification process because the volumetric strain increases when the confining pressure loss increases. The large magnitude of deformation at which the rock reaches its peak stress reflects the extent of deformation. The stress-to-strain ratio in the elastic phase reflects the ability of the rock to resist deformation after it is subjected to stress. As shown in Fig. 29, the elastic modulus of surrounding rock attenuates with increasing wastage rate.

The attenuation formula for elastic modulus and wastage rate is obtained by fitting the scatter points of the elastic phase of the stress–strain curve of surrounding rock. The formula is as follows:

5 $$E = - 0.957 + 2.43e^{ - 0.037d} .$$

By substituting Eq. (4) into Eq. (5), the attenuation formula for the wastage rate and elastic modulus of the surrounding rock can be derived as follows:

6 $$E = - 0.957 + 2.43e^{{ - 0.037e^{\begin{gathered} \left( { - 5.47289 - 0.03994e^{{ - \frac{{\left( {Z - 27} \right)^{2} }}{2.26585}}} } \right) + e^{{ - 8.0221 + 0.24597Z - 0.00392Z^{2} }} t - \hfill \\ \left( { - 0.00002269 + 0.00049686e^{{ - \frac{Z}{6.3552}}} } \right)t^{2} \hfill \\ \end{gathered} } }} ,$$

where *E* is the elastic modulus; *Z* is the axle weight; and *t* is the operating time. Evidently, the wastage rate of surrounding rock is inversely proportional to its elastic modulus. A 5% increase in wastage rate leads to approximately a 25% decrease in elastic modulus. When the surrounding rock does not experience any loss, the initial elastic modulus is 1.46 GPa. However, when the void ratio reaches 15%, the elastic modulus decreases to 0.44 GPa, indicating a 70% reduction. With increasing wastage rate, the decline in the elastic modulus of surrounding rock became more distinct. According to the current “Code for Design of Railway Tunnels” (TB10003-2016), the physical and mechanical parameters of sandstone surrounding rock of different classes and the elastic modulus of Class VI surrounding rock are typically less than 1 GPa. With this as a reference point, when the wastage rate exceeds 7%, the sandstone surrounding rock class transitions from Class V to Class VI.

### Peak strength decay law

**Figure 30:**
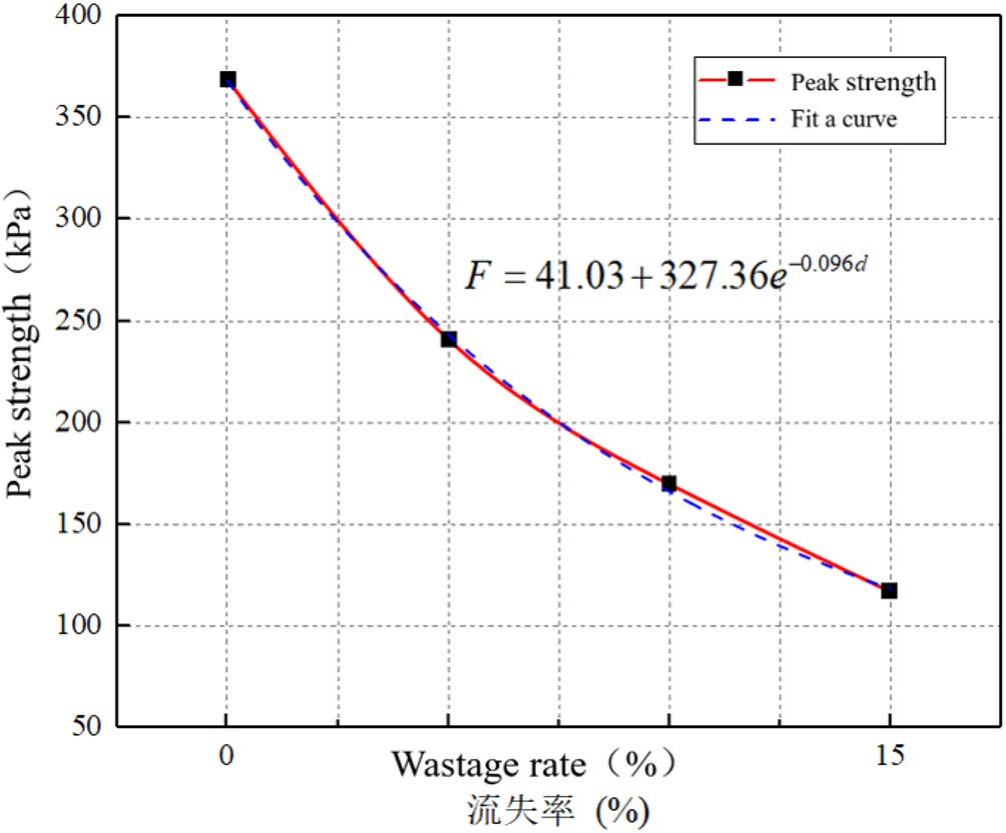
Failure peak strength attenuation curve.

An increase in the wastage rate of surrounding rock leads to the formation of more voids and cracks within the surrounding rock. These cracks cause the uneven stress distribution within the sandstone surrounding rock, subsequently reducing the overall strength of the rock. As the wastage rate increases, the attenuation of the failure peak strength of surrounding rock became more evident, as shown in Fig. 30.

The attenuation formula for the peak strength and wastage rate is derived by fitting the scatter points of the elastic phase of the stress–strain curve of the surrounding rock. The formula used is as follows:

7 $$F = 41.03 + 327.36e^{ - 0.096d} .$$

By substituting Eq. (4) into Eq. (7), the attenuation formula for the wastage rate and peak strength of the surrounding rock can be derived as follows:

8 $$F = 41.03 + 327.36e^{{ - 0.096}{e^{\begin{gathered} \left( { - 5.47289 - 0.03994e^{{ - \frac{{\left( {Z - 27} \right)^{2} }}{2.26585}}} } \right) + e^{{ - 8.0221 + 0.24597Z - 0.00392Z^{2} }} t - \hfill \\ \left( { - 0.00002269 + 0.00049686e^{{ - \frac{Z}{6.3552}}} } \right)t^{2} \hfill \\ \end{gathered} } }}$$

where *F* is the peak strength; *Z* is the axle weight; and *t* is the operating time. As shown in Fig. 30, the degradation pattern of the peak strength of surrounding rock is similar to that of the elastic modulus, exhibiting a relatively rapid deterioration rate of the peak strength before reaching a wastage rate of 7%. When the void ratio reaches 15%, the peak strength is 117 kPa, which is a 68% decrease compared with the state without voids and a 49% decrease compared with the peak strength when the void ratio is 7%.

## Conclusion

This study is based on the long-term monitoring of the contact and water pressures of surrounding rock. Through laboratory experiments, the mechanism and deterioration characteristics of void development in sandy soil surrounding rock were analyzed. From a discrete element perspective, the development pattern and extent of surrounding rock deterioration due to the effects of erosion are examined using PFC. The main conclusions of this study are as follows.

1. Based on the engineering field data collected over a year of operation, the water pressure at the left rail measurement point increased from 117.35 to 286.86 kPa. The soil pressure at the crown measurement point reached a maximum of 181.10 kPa. The bottom surrounding rock continuously deteriorated owing to the combined effects of groundwater and train loading, resulting in a progressive increase in soil and water pressures at the bottom. This phenomenon poses a significant risk of destabilizing the tunnel foundation structure over time. Providing new insights into the effects on long-term stability of tunnels.
2. The establishment of an indoor experimental model enabled the analysis of the void development pattern in the sandy soil surrounding rock. The characteristic manifestation of void development at the base surrounding rock is the formation of large, suspended voids. Under the influence of heavy-haul train loads, the dynamic water flow created a “cone-shaped” void at the bottom of the surrounding rock. Among the effects of train action, dynamic impact on vertical positions is the most evident, providing new experimental support for understanding the voiding mechanism of Sandstone Surrounding Rock.
3. The numerical simulation analysis divides the deterioration process of the base surrounding rock into three characteristic stages. The first stage is the initial operating period when the integrity of the base surrounding rock is relatively satisfactory, closely adhering to the vault structure. However, some voids are formed due to factors, such as construction or geological conditions. The second stage occurs when localized voids occur at the bottom surrounding rock and groundwater continuously erodes the surrounding rock under dynamic action. This results in the partial deterioration of the integrity of the base surrounding rock. The third stage is the complete void formation stage in which the surrounding rock experiences a further decrease in load-bearing capacity and strength under the action of water flow. This causes an imbalance in the stress of the tunnel bottom structure, rendering the structure highly susceptible to defects.
4. Calculations indicated that an increase in wastage rate affected the degradation of the mechanical parameters of surrounding rock. With a 5% increase in wastage rate, the elastic modulus and peak strength decreased by approximately 25% and 45%, respectively. As the wastage rate increased, both the elastic modulus and peak strength gradually decreased. Finally, a degradation formula corresponding to the degradation curve was obtained via fitting, which provides a new numerical method for assessing the stability of sandy soil surrounding rock.

## Data Availability

The datasets used or analysed during the current study are available from the corresponding author on reasonable request.
